# B4GALT5 deficiency impairs glycosphingolipid biosynthesis: a new congenital disorder of glycosylation?

**DOI:** 10.1016/j.jlr.2026.101102

**Published:** 2026-07-09

**Authors:** Linda Montavoci, Michele Dei Cas, Anna Caretti, Omar Ben Mariem, Ivano Eberini, Laura Giaquinto, Alessandro De Falco, Maria Antonietta de Matteis, Nicola Brunetti-Pierri, Marco Trinchera

**Affiliations:** 1 Department of Health Sciences, University of Milan, Milano, Italy; 2 Department of Pharmacological and Biomolecular Sciences "Rodolfo Paoletti", Università degli studi di Milano, Milano, Italy; 3 Department of Molecular Medicine and Medical Biotechnology, Federico II University, Napoli, Italy; 4 Telethon Institute of Genetics and Medicine, Pozzuoli, Naples, Italy; 5 Department of Translational Medicine, Federico II University, Napoli, Italy; 6 Genomics and Experimental Medicine Program, Scuola Superiore Meridionale (SSM, School of Advanced Studies), Naples, Italy; 7 DMC, University of Insubria, Varese, Italy

**Keywords:** brain lipids, CRISPR-Cas9 genome editing, fluorescence microscopy, galactosyltransferase, glycolipids, intellectual disability, lactosylceramide synthase, lipidomics, homology modeling, sphingolipids

## Abstract

Lactosylceramide is a glycosphingolipid precursor synthesized by two dedicated galactosyltransferases, B4GALT5 and B4GALT6. The specific roles of B4GALT5 and B4GALT6 in humans have not yet been clearly defined. Here, we report the first human case with bi-allelic loss-of-function variants in B4GALT5, suggesting that intact B4GALT5 activity is indispensable for normal glycosphingolipid biosynthesis and human development. We identified bi-allelic variants in the *B4GALT5* gene in a child presenting with microcephaly, mild cognitive impairment, and bilateral cataracts. B4GALT5/6 double KO cells transfected with *B4GALT5* carrying either of the variants identified in the patient lacked lactosylceramide synthase activity and failed to produce glycosphingolipids. In silico analyses predicted decreased protein stability and impaired UDP-Gal binding for both B4GALT5 variants. Together, these findings indicate that both variants result in deficient B4GALT5 activity, leaving B4GALT6 as the sole source of lactosylceramide synthase activity. Consistent with this, patient plasma and fibroblasts exhibited an approximately 80% reduction in glycosphingolipid levels compared with healthy controls. Unexpectedly, when expressed in model cells human B4GALT6 displayed lower expression and lower catalytic activity, than human B4GALT5, raising questions about its capacity to compensate for B4GALT5 deficiency. In conclusion, we identified a potential new congenital disorder of glycosylation caused by deficient lactosylceramide synthase activity that may be insufficient to support glycosphingolipids synthesis at levels required for normal brain function.

The first step in biosynthesis of the oligosaccharide chain of mammalian glycosphingolipids is the addition of glucose or galactose to ceramide to form glucosylceramide (GlcCer) or galactosylceramide (GalCer), respectively. While GalCer is a precursor of the end products sulfatide or ganglioside GM4, GlcCer is a precursor for LacCer, which is the substrate for the synthesis of almost all other glycosphingolipid series, including the ganglio-, globo-, and lacto/neolacto-series of glycosphingolipids (1, 2). Addition of β-Gal to the 4-position of Glc in GlcCer is mediated by two β1,4-galactosyltransferases: B4GALT5 and B4GALT6, which share 70.4% sequence identity at the amino acid level. Knockout (KO) of the *b4galt6* gene resulted in viable and phenotypically normal mice, whereas KO of the *b4galt5* gene was embryonically lethal. However, conditional *b4galt5* KO in the central nervous system (CNS) resulted in a normal mouse phenotype, suggesting a compensatory function for *b4galt6* in the mouse brain. By contrast, CNS-specific *b4galt6* and *b4galt5* double KO mice were born normally but showed growth retardation and motor deficits at 2 weeks and died by 4 weeks of age, confirming the important role of *b4galt5/b4galt6* in glycosphingolipid biosynthesis in the brain (3, 4, 5).

The specific roles of B4GALT5 and B4GALT6 in humans have not yet been clearly defined. Here, we report the first human case with bi-allelic loss-of-function variants in B4GALT5, suggesting that intact B4GALT5 activity is indispensable for normal glycosphingolipid biosynthesis and human development.

A young child presenting with mild cognitive impairment, early-onset bilateral cataracts, and microcephaly was investigated by exome sequencing, which identified potentially pathogenic compound heterozygous variants in the *B4GALT5* gene. Although no other cases carrying bi-allelic *B4GALT5* variants were found, we hypothesize that this case may represent a novel and distinct congenital disorder of glycosylation (CDG) involving glycosphingolipid biosynthesis, an expanding group of inherited diseases (2, 6, 7). To investigate this hypothesis, we functionally evaluated the effects of both variants identified in the patient on the enzyme activity and glycosphingolipid profiles. To this end, we generated single and double B4GALT5 and B4GALT6 KO HEK293T cells by CRISPR-Cas9-mediated genome editing. Furthermore, structural analysis of wild-type WT and variant B4GALT5 proteins was carried out in silico, via homology modeling and molecular dynamics simulations. In addition, glycosphingolipid profiles were determined in the serum of the patient and various controls as well as in patient and control fibroblasts under both steady-state and flux conditions. Because defective B4GALT5 could be pathogenic only if B4GALT6 is unable to compensate, several properties of human B4GALT6 were also investigated in parallel experiments.

## Material and methods

### Chemical and reagents

The following materials were purchased: DMEM and FBS from EuroClone Life Science Division; penicillin/streptomycin, Trypsin; protease inhibitors from Roche Italia; SYBR Green system from Takara; ReliaPrep™ Miniprep RNA extraction System and GoScript Reverse Transcription Mix from Promega; synthetic oligonucleotides from Eurofins Genomics.

The chemicals methanol, chloroform, formic acid, acetic acid, ammonium acetate, and ammonium formate, dibutylhydroxytoluene (BHT) were all at analytical grade and purchased from Sigma-Aldrich. All aqueous solutions were prepared using purified water at a Milli-Q grade. Lipid standards were purchased from Avanti Polar (supplied by Sigma-Aldrich).

### Cell culture

HEK293T cells and control fibroblasts were cultured as reported (8). HeLa cells were grown in high-glucose (4,500 mg/l) DMEM supplemented with 10% FBS. Skin fibroblasts from the patient were obtained for diagnostic purposes and made available for research upon written informed consent and approval by the local internal review board (Telethon Foundation, Telethon Undiagnosed Diseases Program). All studies presented in this work comply with the principles of the Declaration of Helsinki.

### Genetic analysis-sequencing

Exome sequencing (WES) did not detect any causative variants in known disease genes, but it revealed two missense variants in the *B4GALT5* gene, validated by Sanger sequencing, and each variant was inherited from one parent: the p.(Arg173His) variant (NM_004776: c.518G>A) and the p.Gly274Arg (NM_0044776: c.820G>A).

### Plasmid constructs

The coding sequences of human B4GALT5 and B4GALT6 were obtained by PCR using total RNA extracted from Huh-7 or Hep-3B cells in the presence of Phusion Taq polymerase (Thermo Fisher Scientific) and a specific primer pair (Supplemental Table S1) containing restriction sites for *Hind*III/*Sfg*I in the forward primer and *Pme*I/*Xba*I in the reverse primer (underlined). Amplification program was as follows: initial denaturing at 98°C for 30 s, 30 cycles at 95°C for 8 s, 64°C for 20 s, 72°C for 45 s, and final extension at 72°C for 10 min. Mouse b4galt6 cDNA was obtained as above using RNA extracted from Neuro2a cells and the proper primer pair with identical restriction sites (Supplemental Table S1). The obtained fragments were cloned in pCDNA3 vector, expanded, and sequenced as reported (8), obtaining plasmids named pCDNA3-B4GALT5, pCDNA3-B4GALT6, and pcDNA3-mb4galt6. pcDNA3-B4GALT5 plasmid was mutated using QuikChange II-E Site-Directed Mutagenesis Kit and the reported primer pairs (Supplemental Table S1), designed to introduce in the sequence the c.518G>A and c.820G>A mutations found in the patient and giving rise to p.R173H and p.G274R enzyme variants, respectively. For cloning B4GALT5 into pCNA4, the gene sequence was amplified by RT-PCR from total RNA extracted from HeLa cells (Supplemental Table S1). The resulting amplicon was cloned into the *EcoR*I and *EcoR*V sites of pCNA4 in-frame with a C-terminal Myc-tag. Using the *Hind*III and *BstEI*I sites present in pCNA4, human and mouse B4GALT6 cDNAs were also cloned in such vector to obtain c-Myc tag at the C-terminus (pCNA4-B4GALT6 and pCNA4-mb4galt6, respectively). To this aim, cDNAs were amplified by PCR using pcDNA3-B4GALT6 or pcDNA3-mb4galt6 as templates (10 ng of each plasmid in 50 μl reaction volume), respectively, using the same forward primers as above and the reverse primers designed to skip the stop codon and to introduce the *BstE*II site (Supplemental Table S1). Amplification conditions were those used for cloning in pcDNA3, but only 22 PCR cycles were employed. The obtained fragments were purified, digested, and cloned in pCNA4 vector that was previously digested with *Hind*III and *BstE*II and gel purified, as described for cloning in pcDNA3. Patient mutations were then introduced in pCNA4-B4GALT5 by site directed mutagenesis as described earlier in pCDNA3. Moreover, WT and variant B4GALT5, as well as human B4GALT6, were subcloned in pFN21 vector (Promega) using the *Sfg*I and *Pme*I sites present in the original cloning primer pairs (Supplemental Table S1, double underlined), as previously reported (9). In this way, cDNAs were placed in frame with Halo tag at the N-terminus.

### Chimeras

Chimeric constructs of human B4GALT6 were prepared starting from pCNA4-B4GALT6 using the *Mlu*I site present in the CMV promoter and the *Xho*I site located at nucleotide 142 of B4GALT6 coding sequence, involving codons 48–50. Upon digestion with both enzymes and agarose gel purification, a fragment encompassing the bulk of the vector and B4GALT6 cDNA was obtained, presenting *Mlu*I and *Xho*I adhesive ends. pCDNA3-B4GALT5 plasmid was used as a template for amplifying the 142 bp sequence representing the first 48 codons of human B4GALT5, using the reported primer pair (Supplemental Table S1), each one containing the *Mlu*I and *Xho*I sites, respectively (underlined). Reaction was performed in the presence of Phusion hot start high fidelity Taq polymerase (Thermofisher) according to the vendor’s protocol. Amplification program was as follows: initial denaturing at 98°C for 30 s, 22 cycles at 95°C for 8 s, 64°C for 20 s, 72°C for 25 s, and final extension at 72°C for 10 min. The amplified fragment was digested by *Mlu*I and *Xho*I, spin-column purified, and ligated to the gel-purified linear plasmid with the corresponding ends. The obtained construct was sequenced, and the translated sequence is shown in Supplemental Fig. S1A. The first 46 amino acids of the chimera, named chi46-T5/T6, correspond to B4GALT5 protein sequence (encompassing the cytosolic tail and transmembrane domain), amino acids 47 and 48 are in common between B4GALT5 and B4GALT6 (QA, underlined in Supplemental Fig. S1A), while the sequence proper of B4GALT6 begins at amino acid 49.

Additional chimeras were constructed inserting a *BspE*I site (TCCGGA) in pCNA4-B4GALT6 through site directed mutagenesis at position 288 of the coding sequence, changing codon 96, CTC, to CTT, still coding lysine. To this purpose, mutagenesis was performed using QuickChange II kit (Agilent) with the oligonucleotides shown in Supplemental Table S1 according to our previous report (8).

Plasmid pCNA4-B4GALT6 (c.288C>T) was digested with *Mlu*I and *Bsp*EI, and the longer fragment isolated by agarose gel electrophoresis. pCDNA3-B4GALT5 plasmid was used as template for amplifying the 308 bp sequence encompassing the first 103 codons of human B4GALT5 using the proper primer pair (Supplemental Table S1), containing the *Mlu*I and *BspE*I sites, respectively (underlined). Amplification was performed as above reported for chimera chi46-T5/T6. The amplified fragment was digested by *Mlu*I and *BspE*I, spin-column purified, and ligated to the linear plasmid gel purified upon digestion with *MluI* and *BspE*I. The obtained construct was sequenced and the translated sequence was found to be as reported in Supplemental Fig. S1A. The first 101 amino acids of the chimera, named chi104-T5/T6, correspond to B4GALT5 protein sequence (encompassing cytosolic tail, transmembrane domain and stem region), amino acids 102–104 (LPE, underlined) correspond to amino acids 96–98 of B4GALT6, and are in common between B4GALT5 and B4GALT6, while the sequence proper of B4GALT6 begins at amino acid 105 that corresponds to amino acid 99 of the original B4GALT6 coding sequence. pCDNA3-mb4galt6 plasmid was used as a template for amplifying the 290 bp sequence encompassing the first 96 codons of mouse b4galt6 using the primer pair reported (Supplemental Table S1) containing the *Mlu*I and *BspE*I sites (underlined). Amplification and cloning were performed as for chi104-T5/T6. The obtained construct was sequenced and the translated sequence is shown in Supplemental Fig. S1A. The first 85 amino acids of the chimera, named chi-97mt6/T6, correspond to mouse b4galt6 protein sequence (comprising cytosolic tail, transmembrane domain, and stem region), amino acids 86–97 (SDYLVQTTTYLP, underlined) are in common between mouse and human B4GALT6, while the sequence proper of human B4GALT6 begins at amino acid 98 of the human coding sequence. The stem-region sequences are compared at the amino acid level in Supplemental Fig. S1B, and the corresponding cDNA sequences are shown in Supplemental Fig. S1C. The complete cDNA sequences of all constructs reported in this article are presented in Supplemental Fig. S2.

### Cell manipulation

HEK293T cells and the obtained KO clones were transiently transfected using Fugene HD (Promega), as previously reported (8). At different times upon transfection, cells were trypsinized and divided into different aliquots: one for RNA extraction by the Relia-Prep RNA kit (Promega), one for sphingolipid quantification by LC-MS/MS, one for preparing homogenates (0.1 M Tris/HCl buffer, pH 7.5, containing 0.5% Triton X-100) to be used as the enzyme source for in vitro assays, and the last one for protein extraction by RIPA buffer to be analyzed by western blotting. All samples were stored at −80 °C until further analysis.

### CRISPR-Cas9 genome editing

CRISPR/Cas9-mediated knockout of B4GALT5 and B4GALT6 genes was performed using the corresponding CRISPR/Cas9 KO and HDR plasmids (Santa Cruz Biotechnology, sc-406387-KO/HDR for B4GALT5 and sc-411270-KO/HDR for B4GALT6), which introduce puromycin resistance and red fluorescent protein (RFP) cassettes at multiple Cas9 cleavage sites. HEK293T cells (0.1 × 10^6^ in a well of a 48-wells plate) were transfected with equal amounts (0.2 μg) of each plasmid using Fugene-HD. Two days after transfection, cells were trypsinized, plated at different dilutions, and allowed to grow under puromycin selection (2 μg/ml in regular growing medium). Individual red fluorescent colonies were isolated by cloning cylinders and expanded. RNA from each clone was extracted, submitted to RT-PCR using the same primers and conditions used for initial cloning, but repeating 38 PCR cycles, and analyzed by 1% agarose gel electrophoresis. The absence of full-length B4GALT5 or B4GALT6 transcripts found in the parental HEK293T cells was assumed to be the proof of gene disruption. True KO clones were further characterized by enzymatic assays and GSL profiling (LacCer, GM3, Gb3, HexCer) via LC–MS/MS, as previously described. Two characterized B4GALT5 KO clones were subsequently transfected with a Cre recombinase plasmid (Santa Cruz Biotechnology, sc-418923) to excise the RFP and puromycin resistance cassette. To this aim, B4GALT5 KO clones were plated, transfected, and the day after trypsinized and plated at multiple dilutions as described for the CRISPR/Cas9-HDR plasmids, but allowing the cells to grow with regular DMEM without puromycin. Colonies that lost fluorescence were selected and transferred by cloning cylinders, expanded, and split to treat an aliquot with puromycin. Those who become puromycin-sensitive were selected and then used for the B4GALT6 knockout using the same CRISPR/Cas9 and HDR plasmids and procedures described above.

### Sphingolipid profile

Sphingolipids were extracted from cells (25 μl of plasma; 50 μg of proteins for fibroblasts; less than 200 μg for HEK293T) by using a mixture of water/methanol/chloroform (1:6:3) coupled to alkaline methanolysis (8). The internal standard mixture consisted of GlcCer 12:0, LacCer 12:0 (50 pmol), and GM1a 17:0 (20 pmol), added to each sample before extraction to enable quantitative LC-MS/MS analysis of glycosphingolipids. Five microliters of methanol extract were directly injected into LC-MS/MS. The LC-MS/MS consisted of a by LC Dionex 3000 UltiMate (ThermoFisher Scientific) coupled to a tandem mass spectrometer AB Sciex 3200 QTRAP (AB Sciex, Concord) equipped with electrospray ionization TurboIonSpray™ source operating in positive mode (ESI+). The instrument parameters were: CUR 25, GS1 45, GS2 50, capillary voltage 5.5 kV and source temperature 300°C. Spectra were acquired by multiple reaction monitoring, scanning for protonated molecular ions [M+H]+ of each sphingolipid (Supplemental Table S2). Compound specific conditions were the following: HexCer DP 40, CE 50 eV; LacCer DP 50, CE 50 eV; GM3 DP 60, CE 60 eV and Gb3 DP 60, CE 60 eV. The collision energy was optimized on the prominent daughter ion which is the sphingoid base fragment *m/z* 264.3. Chromatographic separation was achieved on a reverse-phase Acquity BEH C8 column 1.7 μm, 2.1 × 100 mm (Waters, MA, USA) equipped with pre-column using as mobile phases (A) water + 0.2% formic acid + 2 mM ammonium formate and (B) methanol + 0.2% formic acid + 1 mM ammonium formate. The flow rate was 0.3 ml/min and the column temperature was 30°C. The elution gradient (%B) was set as below: 0–3 min (80–90%), 3.0–6.0 min (90%), 6.0–19.0 min (90–99%), 19.0–20.0 min (99–80%), held until 24 min. Due to lack of authentic standards with every fatty acid chain, those which are not available were quantified as a reference of the closest sphingolipids subspecies.

### Metabolic labelling of glycosphingolipids using ^13^C_6_ galactose

Fibroblasts were plated in 12-well plates (0.1 × 10^6^ cells per well) and the day after medium was replaced by fresh regular medium (DMEM containing glucose 25 mM) supplemented with 2.5 mM ^13^C_6_-galactose. Attempts to use low glucose or glucose free DMEM resulted in much lower labeling. After 7–38h, cells were harvested by trypsinization, washed with PBS twice, and pelleted for further analysis. Sphingolipids were extracted (30–50 μg of proteins) and analyzed as reported above except for mass spectrometry conditions that were modified to look for ^13^C_6_ galactose labelled GSLs. The molecular ions [M+H]+ of labelled glycosphingolipids (Supplemental Table S3) were calculated using mass shift + 6.024 Da for hexosylceramides; m/z +12.048 Da for lactosylceramide and GM3; m/z +18.072 for Gb3, Lc3, Lc4 and GM1a. MS/MS fragments specific for labelled hexose incorporation corresponds to labelled HexCer moieties such as m/z 688.58–856.65 (C16) and 800.70–968.77 (C24). Lc3 and GM1a revealed also the MS/MS fragment *m/z* 372.16 which corresponds to ^13^C-hexose + n-Acetyl-hexosamine. The quantification of Lc3, Lc4, and GM1a was not included in this study.

### Metabolic labeling of sphingolipids using ^3^H-sphingosine

Fibroblasts were plated in 12-well plates, and the following day the medium was replaced with fresh DMEM supplemented with [^3^H]-sphingosine for 1 or 2 h. Cells were then washed with PBS, collected by trypsinization, and processed for lipid extraction. Lipid extracts were dissolved in CHCl_3_ and loaded on TLC silica gel plates.

### LacCer synthase activity

Enzymatic activity was measured using deuterated GlcCer (GlcCer-d7, C15 Glucosyl(β)ceramide-d7) as substrate, and by quantifying the formation of deuterated LacCer (LacCer-d7, C15 Lactosylceramide-d7) as previously described (10). LC-MS/MS analysis was performed in multiple reaction monitoring mode, monitoring the transition m/z 693.60>271.30 (DP 40 V, CE 50 eV) for GlcCer-d7 and m/z 855.60>271.30 (DP 50 V, CE 50 eV) for the product LacCer-d7. For a graphical description and apparent kinetic constant (K_m_) calculations, the Hanes–Woolf equation was used.

### Reverse transcription quantitative real-time PCR

First strand cDNA was synthesized from 0.5 to 1 μg of total RNA by a commercial kit and 1 μl of first strand reaction was amplified in a volume of 20 μl using Sybr Premix Ex Taq, ROX as the reference dye, and a StepOnePlus instrument as previously reported (11). The temperature of annealing was 60°C. The amplified target cDNAs were quantified as ΔCt with respect to GAPDH.

### Western blotting

Aliquots of protein extracts (0.5–40 μg) from each sample were separated on 10 or 12% SDS-PAGE gel and transferred onto a nitrocellulose membrane via electroblotting. Following TBS-T (Tris-HCl buffer saline with Tween) washes and incubation with 5% nonfat dry milk for 1 h at room temperature, the membrane was incubated overnight at 4°C with anti-c-Myc (Santa-Cruz, 1:2000) or anti-HaloTag (Promega, 1:1000) primary antibody. The membrane was further blotted with horseradish peroxidase-conjugated secondary antibody (1:10,000, anti-mouse or anti-rabbit, respectively, Jackson Lab). The protein bands were detected and quantified by chemo-luminescent method by the Alliance™ UVITEC system which provided the band intensity quantification as well. Every membrane was stripped and incubated with β-actin as previously reported (8).

### Immunofluorescence and confocal microscopy

HeLa cells and fibroblasts were transfected using TransIT-LT1 reagent (Mirus Bio LLC) according to the manufacturer’s instructions and allowed to express for 24 h prior to processing. Cells were fixed with 4% PFA for 10 min at room temperature, washed with PBS, and incubated in blocking solution for 30 min. The blocking solution consisted of 0.05% saponin, 0.5% BSA, and 50 mM NH_4_Cl in PBS. For plasma membrane staining, cells were incubated in the same buffer lacking saponin.

Primary antibodies were diluted in blocking solution and incubated with the samples for 60 min at room temperature, followed by incubation with appropriate Alexa Fluor–conjugated secondary antibodies (Invitrogen) for 30 min. The following primary antibodies were used: goat polyclonal anti-FLAG (Bethyl, A190-101A), mouse monoclonal anti-c-Myc (Santa Cruz, sc-40), mouse monoclonal Golgin97 (ThermoFisher, A-21270), sheep anti-TGN46 (BioRad #AHP500), mouse monoclonal anti-EEAI (BD Transduction Laboratories™, 610457), and rabbit polyclonal anti-LAMPI (ABCAM, ab241070). Rabbit polyclonal antibodies against Golgin97, GM130, and VAP-A were generated in-house. Plasma membrane Gb3 was visualized using Cy3-conjugated Shiga toxin subunit B (StxB) (12).

For HaloTag labeling experiments, fibroblasts transfected with Halo-tagged B4GALT5 were incubated with 1μM TMR ligand (Promega, G8252) for 15 min at 37°C, washed three times in DMEM, and further incubated in fresh medium for 30 min at 37°C prior to fixation. Coverslips were mounted using Mowiol mounting medium. Images were acquired using a Zeiss LSM800 confocal microscope and processed with Fiji (ImageJ; National Institutes of Health) and Adobe Photoshop software.

### Statistical analysis

Statistical analyses were performed using GraphPad Prism version 8. Data are presented as mean ± SD. For the comparison of B4GALT5 and B4GALT6 mRNA transcript levels within each sample, paired *t* test was used. For glycosphingolipid profiles and enzyme activity assays, statistical comparisons among three or more groups were performed using one-way ANOVA followed by Tukey’s post hoc test for pairwise comparisons or Dunnett’s post hoc test for comparisons against the patient. Statistical significance for mRNA analyses is indicated using the symbol (#), whereas significance for glycosphingolipid profile and enzyme activity analyses is indicated using (∗) or ($), as specified in the corresponding figure legends.

### Homology modeling

Since there were no experimental structures of these enzymes, the 3D models were predicted via homology modeling, using as template the X-ray structure of β-1,4 Galactosyltransferase 1 catalytic domain, PDB code: 2FYB (13). The sequence identity between B4GALT5 and the template was 46.15% (similarity: 61%). The patient’s mutations were introduced into the wild-type (WT) B4GALT5 structure, generating two distinct mutant models. The homology modeling was performed with Prime (Prime, Schrödinger, LLC, New York, NY, USA, 2024). For each model, only the catalytic luminal domain (residues 110 to the C-terminus) was modelled, since the structural template comprises only the luminal domain and all investigated mutations are located within this region. All models included the uridine-diphosphate (UDP) moiety derived from the 2FYB crystal structure. The UDP-α-D-galactose (PubChem ID: 18068) was prepared and aligned onto the UDP to generate the initial complexes for molecular dynamics simulations.

As an initial assessment of the impact of these mutations, the ΔStability for each B4GALT5 variant was calculated. This value is defined as the difference between the free energy of the mutant protein and that of the wild-type protein: ΔStability = ΔG_mut − ΔG_wt. Since higher free energy corresponds to lower protein stability, higher positive values indicate greater destabilizing effects. For these calculations, mutations associated with ΔStability values greater than the threshold of 3 kcal/mol were considered to have a significant impact on protein stability (14).

### Molecular dynamics simulation

All the systems for MD simulations were prepared using the Desmond (Schrödinger Release 2024-2: Desmond Molecular Dynamics System, D. E. Shaw Research, New York, NY, 2024. Maestro-Desmond Interoperability Tools, Schrödinger, New York, NY, 2024) System Builder tool as previously described (15). The system was solvated with SPC water in a box extending 10 Å beyond the protein. Na^+^ and Cl^-^ ions were added to neutralize the charge and reach 0.15 M. MD simulations were run using Desmond and parametrizing the system with the OPLS4 force field (16), periodic boundary condition (PBC) were applied, a temperature of 300 K (Nose-Hoover thermostat), a pressure of 1 bar (Martyna-Tobias-Klein barostat), a 2 fs timestep, a 0.5 ps frame interval, for 100 ns in total for each simulation.

For each trajectory, the Root Mean Square Deviation (RMSD) and the Root Mean Square Fluctuation (RMSF) were calculated on α carbons using the Schrödinger API. Cluster analyses were also performed according to the GROMOS (17) method, using as the cluster distance the RMSD matrix of α carbons with respect to the first frame of the MD simulation (15). The threshold was set to 2 Ä and the representative structures of the most populated clusters were chosen for the subsequent analyses. The interactions between the protein and the ligand were calculated via the Schrödinger API, setting a threshold of 30% of simulation time for significance.

## Results

### Case report

A 6 year and 3-month-old Caucasian girl was evaluated for bilateral cataract, subluxation of the left eye lens, attention-deficit/hyperactivity disorder, and microcephaly. The proband was the third child of non-consanguineous healthy parents and she was born at term of unremarkable pregnancy, with normal perinatal history and a birth weight of 3,100 g (−0.8 SD). She spoke her first words at 12 months of age, but she achieved independent walking at 24 months. She had speech delay, learning difficulties, and attention-deficit/hyperactivity disorder. At 4 years of age an ophthalmological evaluation revealed bilateral lens opacities and lens subluxation in the left eye. She underwent bilateral lens extraction and artificial lens implantation. At her last evaluation, at the age of 7 years and 10 months, her weight was 30 kg (+0.1 SD), her height was 123.5 cm (+0.7 SD), and her occipitofrontal circumference was 48.5 cm (−1.3 SD). She was noted to have long philtrum and prognathism. She was evaluated by echocardiogram that did not reveal any cardiac defect. Galactose-1-phosphate uridylyltransferase (GALT) activity and total blood galactose concentrations in blood were within the normal range. Array-based comparative genomic hybridization showed microdeletions of 5q21.2 and 7p21.2 of 83 Kb and 10.6 Kb respectively; both are found in control individuals and were interpreted as benign copy number variants. Exome sequencing revealed compound heterozygous variants in *B4GALT5*: c.518G>A-p.R173H and c.820G>A-p.G274R, both classified as likely pathogenic based on American College of Medical Genetics and Genomics criteria (PM2, PP3). The p.(R173H) variant is not present in gnomAD, has a Combined Annotation Dependent Depletion (CADD) (http://cadd.gs.washington.edu) score of 32, and it is not annotated in ClinVar. The p.(G274R) variant has an allele frequency of 3.977 × 10^−6^ in gnomAD (1 of 251,428 alleles, observed only in the heterozygous state), a CADD score of 26.4, and is not annotated in ClinVar. This case was included in DECIPHER as Patient ID: 412221.

We set out to investigate the expression and enzymatic activity of the identified B4GALT5 variants in a B4GALT5/6 double KO cell model.

### Analysis of patient plasma and fibroblasts in culture

**Fig. 1:**
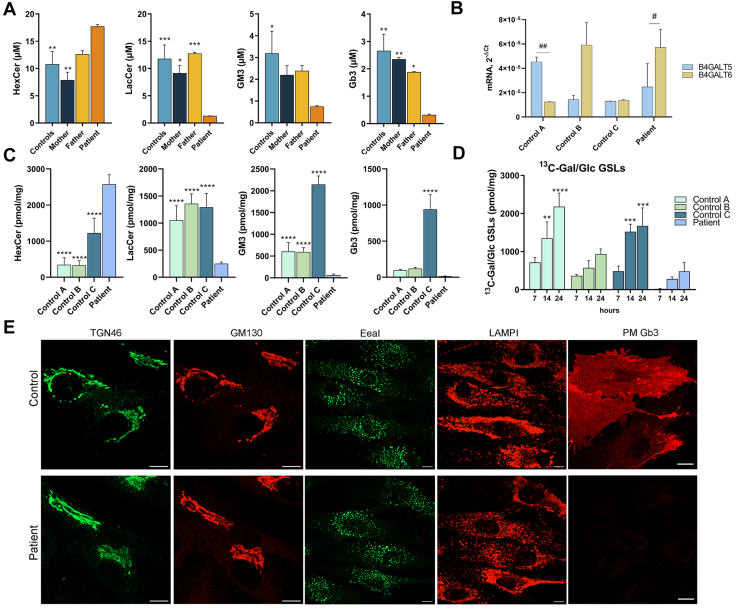
Analysis of patient plasma and fibroblasts in culture. A: Patient plasma was extracted and analyzed by LC-MS/MS together with plasma from her mother, father, and five age-matched healthy controls. In this panel, glycosphingolipids are reported as the sum of individual species with fatty acyl chains ranging from C16:0 to C24:0 and C24:1. Statistical comparisons (∗) were performed between the patient and each individual control, including mother and father. B: Dermal fibroblasts from six healthy individuals and from the patient were grown in culture and the total RNA extracted, reverse transcribed and the cDNA amplified by qPCR using primers specific for B4GALT5 and B4GALT6, plus GAPDH used as the reference housekeeping gene. Three control fibroblasts showing different ratios between the levels of B4GALT5 and B4GALT6 transcripts were shown here (see Supplemental Fig. S3 for the others). Statistical significance (#) refers to the comparison between mRNA of B4GALT5 and B4GALT6 within each sample. C: The GSL profile was determined by LC-MS/MS in the same fibroblasts shown in B. Values are the mean ± SD for four replicates of each fibroblasts grown independently. In this panel, glycosphingolipids are reported as the sum of individual species with fatty acyl chains ranging from C16:0 to C24:0 and C24:1. Statistical significance (∗) was assessed by comparing patient with each control fibroblast line (A–C), which are shown separately rather than as a mean value in order to highlight inter-individual variability. D: The same fibroblasts as in B and C were grown in the presence of ^13^C_6_-galactose for the indicated times and the GSL containing ^13^C_6_-galactose and ^13^C_6_-glucose residues quantitated by LC-MS/MS. The levels of the main GSLs that incorporated ^13^C_6_-galactose and/or ^3^C_6_-glucose, LacCer, GM3 and Gb3, are presented as their sum (C16 + C24) due to the precursor-product relationship between LacCer and both GM3 and Gb3. Values are the mean ± SD for three independent incubations. Statistical analysis (∗) was performed by comparing the patient with each individual control (A–C) at the same incubation time. E: Control and patient fibroblasts stained with various subcellular compartment markers. From left to right: Golgi apparatus markers TGN46 (green) and GM130 (red), endosomal marker EEA1 (green), and lysosomal marker LAMP1 (red). The right panel shows plasma membrane Gb3 visualized by StxB-Cy3 staining of non-permeabilized cells. Scale bars, 10 μm. In Figure 1 statistical significance was evaluated by one-way ANOVA followed by Dunnett’s post hoc test. ∗*P* < 0.05, ∗∗*P* < 0.01, ∗*P* < 0.005, ∗∗*P* < 0.001 versus patient. Paired *t* test was used to assess differences between B4GALT5 and B4GALT6 mRNA levels within each sample. #*P* < 0.05, ##*P* < 0.01.

To further evaluate the potential effects of the identified *B4GALT5* variants, we performed glycosphingolipid analyses of plasma samples and primary cultured fibroblasts obtained from the patient. Plasma samples from the patient’s parents and five age-matched healthy individuals were used as controls. Significant differences in the amounts of circulating LacCer and Gb3 were observed, with more than a 5-fold reduction in LacCer and Gb3 levels in plasma samples from the patient compared with all controls. Although they did not reach statistical significance, circulating GM3 concentrations were also reduced in patient plasma, whereas hexosylceramide (HexCer) concentrations were increased compared with controls (Fig. 1A). In cultured primary fibroblasts obtained from six healthy controls, the levels of B4GALT5 and B4GALT6 transcripts varied among samples. However, in most fibroblast samples, including the patient fibroblasts, B4GALT6 transcript levels were 3- to 8-fold higher than those of B4GALT5, as determined by RT-qPCR (Supplemental Fig. S3). To determine whether such a B4GALT5/6 transcript ratio affects GSL expression, we selected three control fibroblast lines for subsequent experiments in which B4GALT6 transcript levels were similar to, lower than, or higher than those of B4GALT5 (Fig. 1B). Compared with these three controls, the patient sample showed a marked reduction in LacCer, GM3 and Gb3 levels, together with an accumulation of HexCer (Fig. 1C). These differences reached statistical significance, despite the variable GSL profiles observed among control fibroblasts. The same fibroblasts were also incubated for different periods in the presence of ^13^C_6_-galactose, and the profiles of GSLs containing ^13^C_6_-galactose and/or ^13^C_6_-glucose were determined (Fig. 1D). Efficient metabolic conversion of ^13^C_6_-labeled galactose into ^13^C_6_-labeled glucose has previously been observed (18). Analysis of glycosphingolipids (LacCer and GM3) revealed that less than 30% of the total labeled species contained only the ^13^C_6_-galactose residue, resulting in a mass-to-charge ratio increase of +6.02. By contrast, more than 70% of the labeled species incorporated both ^13^C_6_-galactose and ^13^C_6_-glucose residues, as reflected by a combined mass-to-charge shift of +12.02. Although the rate of GSL synthesis varied among individual control fibroblast lines, GSL synthesis was delayed in the patient’s fibroblasts, which were unable to synthesize the expected amounts of GSLs.

The sphingolipid neosynthesis profile of the patient’s fibroblasts was further validated by labeling the cells with [^3^H]-sphingosine for short pulse periods (1 and 2 h). Levels of GSLs downstream of LacCer in the biosynthetic pathway were reduced in patient fibroblasts compared with control cells, whereas an accumulation of HexCer was observed (Supplemental Fig. S4). Quantitative analysis of the sphingolipid profile after 1- and 2-h [^3^H]-sphingosine pulses is presented in the same figure (Supplemental Fig. S4).

A general characterization of subcellular compartments revealed no detectable differences between patient and control fibroblasts. In particular, Golgi apparatus morphology (TGN46 and GM130 staining), endosomal compartments (EEA1 staining), and lysosomes (LAMP1 staining) were comparable between the two groups (Fig. 1E). Similarly, no evident alterations were observed in endoplasmic reticulum organization (VAPA staining) or actin cytoskeleton organization, as assessed by phalloidin staining (data not shown). By contrast, analysis of Gb3 at the plasma membrane by Stx-Cy3 staining revealed that patient fibroblasts lacked detectable Gb3 (Fig. 1E).

### Characterization of HEK293T KO model

**Fig. 2:**
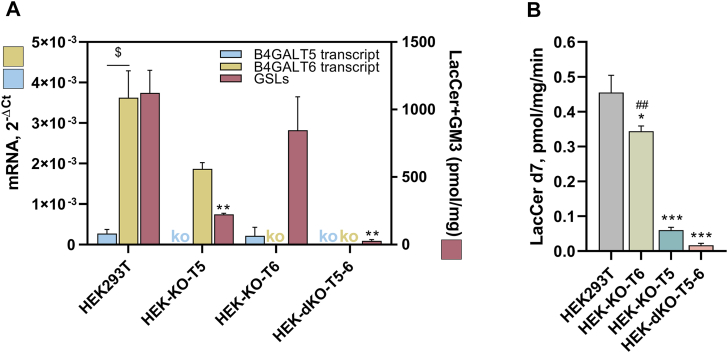
Characterization of KO HEK293T cell model. A: HEK293T cells lacking B4GALT5 (HEK-KO-T5), B4GALT6 (HEK-KO-T6), or both (HEK-dKO-T5-6) were subjected to RNA and GSL (LacCer + GM3) quantification by RT-qPCR and LC-MS/MS, respectively. LacCer + GM3 values represent the sum of all acyl species from C16:0 to C24:0 and C24:1. DCt refers to the difference between target and GAPDH transcripts. Values labeled as ko are for transcripts in the respective KO clones, which were not suitable because the CRISPR-Cas9 procedure employed (see Materials and methods) may have removed or not the annealing sequence that the PCR primers were designed to bind to from the target gene. Cellular levels of relevant GSLs produced via LacCer synthase activity (LacCer and GM3) are reported together, because of their precursor-product relationship. Values are the mean ± SD for four replicates of a single clone grown independently. On the left axis, statistical significance refers to the comparison between B4GALT5 and B4GALT6 mRNA levels within HEK293T cells, indicated by (#). On the right axis, it is highlighted the comparison of GSL levels in each KO clone with parental HEK293T cells (∗). B: Homogenates obtained from parental HEK293T cells and the same KO clones described in panel A were evaluated for C15:0 LacCer d7 synthase activity in vitro, using C15:0 GlcCer d7 as an acceptor substrate and UDP-Gal as a donor. The amount of LacCer d7 formed was quantified by LC-MS/MS. Values are the mean ± SD for two independent assays performed in duplicates. Statistical significance refers to the comparisons against parental HEK293T cells (∗), and to the indicated comparison with HEK-dKO-T5-6 ($). In Figure 2 statistical significance was evaluated by one-way ANOVA followed by Dunnett’s post hoc test in panel A and Tukey’s post hoc test in panel B. ∗∗*P* < 0.01 ∗∗∗*P* < 0.005 versus HEK293T; $$ *P* < 0.01 versus HEK-dKO-T5-6. Paired *t* test was used to assess differences between B4GALT5 and B4GALT6 mRNA levels within each sample. #*P* < 0.05.

Following a published genome editing procedure, we obtained several cell clones that were puromycin-resistant and expressed RFP, indicating that the relevant gene cassette had been correctly inserted into the *B4GALT5* or *B4GALT*6 locus, thereby preventing expression of the corresponding protein. Because antibodies specific to either protein were unavailable, it was not possible to directly confirm loss of expression by western blotting. Instead, we amplified cDNA obtained from the clones using primers specific for the full coding sequences and compared the resulting PCR products with those obtained from the parental cell line. Clones lacking B4GALT5-or B4GALT6-specific cDNA products of the expected size were selected for further experiments (Supplemental Fig. S5). In addition, two *B4GALT5* KO clones were treated with Cre recombinase, through transient transfection of a plasmid encoding Cre recombinase cDNA. B4GALT5 KO clones that lost the RFP/puromycin resistance gene cassette were then selected for a second round of genome editing, to generate B4GALT5/B4GALT6 double KO clones, following the same approach used to generate the B4GALT5 KO HEK293T cells. The resulting B4GALT5/B4GALT6 double KO clones (Supplemental Fig. S5) were isolated and selected as described above and used for further experiments. Comparisons of mRNA levels, glycosphingolipid profiles (Fig. 2A), and LacCer synthase activity (Fig. 2B) between parental HEK293T cells and KO HEK293T clones lacking either B4GALT5 or B4GALT6 (HEK-KO-T5 and HEK-KO-T6, respectively), showed that the parental HEK293T cell line expresses approximately 10-fold higher levels of B4GALT6 mRNA than B4GALT5 mRNA. The parental cells also contained substantial amounts of LacCer and GM3, which together constituted the majority of glycosphingolipids detected by LC-MS/MS. Smaller amounts of other glycosphingolipids, including Lc3 (0.7%), Lc4 (7%), GM1a (1.6%) and Gb3 (1.8%), were also detected; together, these accounted for less than 12% of the total GSL content and were not considered further. Although HEK-KO-T5 cells expressed normal levels of B4GALT6 mRNA, they exhibited an approximately 80% reduction in GSL levels and LacCer synthase activity compared with the parental HEK293T cells. By contrast, HEK-KO-T6 cells maintained a GSL profile similar to that of the parental HEK293T cells, and their LacCer synthase activity was largely unaffected. However, the B4GALT5/B4GALT6 double KO cells (HEK-dKO-T5-6) showed almost complete loss of GSLs (<1% of parental levels) and LacCer synthase activity. Based on these characteristics, HEK-dKO-T5-6 cells appeared to be an ideal host cell line for functional characterization of the *B4GALT5* variants identified in the patient. To verify the reproducibility of these findings, sphingolipid concentrations were measured in cells cultured in multiple independent batches on different days. This approach accounted for potential batch-to-batch variability in culture conditions, media composition, and metabolic states, ensuring that the observed patterns were consistent and not artifacts of a single experiment (data not shown).

### Modeling of B4GALT5 variants

**Fig. 3:**
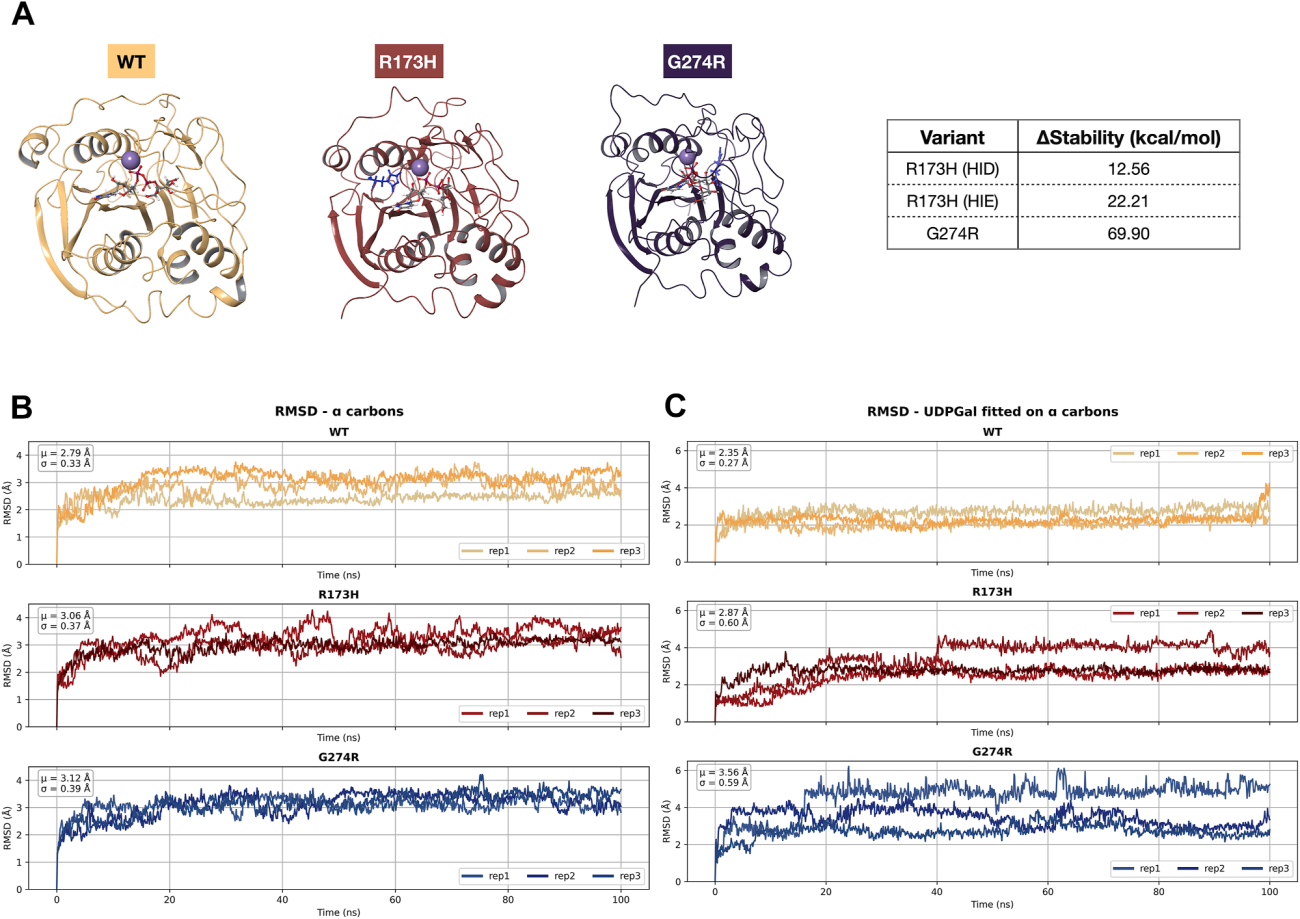
In silico structural analysis. A: On the left side, 3D models containing a Mn2+ ion and UDP-Gal are represented. In R173H model, His173 is highlighted (blue). In G274R model, Arg274 is highlighted (blue). On the right side, ΔStability values are expressed as kcal/mol. For the R173H variant, values for both δ- and ε-protonation of His are reported. B: RMSD values of protein backbone were calculated throughout the MD simulation, and the mean (μ) and standard deviation (σ) are reported. For each protein, one line per replica is shown on the graph. C: RMSD values of UDP-Gal were calculated throughout the MD simulation, and the mean (μ) and standard deviation (σ) are reported. For each protein, one line per replica is shown on the graph.

To provide a framework for assessing the effects of the patient-identified missense variants on B4GALT5 at the structural level, homology modeling was conducted. Homology models of WT and both variant B4GALT5 proteins were generated, and 3D models containing UDP-Gal were obtained using the coordinates of UDP in PDB entry 2FYB as a reference (Fig. 3A). The ΔStability value for each B4GALT5 variant was calculated as shown in Figure 3A, both variants were associated with positive ΔStability values, indicating a negative effect on protein stability.

Molecular dynamics (MD) simulations were then performed to investigate protein behavior in a biologically relevant environment. Because only the luminal domain was modeled, the simulations were carried out in solvent. For each model, three independent 100-ns replica MD simulations were performed. Following the simulations, root-mean-square deviations (RMSDs) were calculated from the resulting trajectories, as an indicator of protein stability. RMSD analyses conducted over 100 ns of MD simulations comparing WT and the two mutants (R173H and G274R) in complex with UDP-Gal; revealed notable differences in structural stability and ligand retention. RMSD values for protein backbone Cα atoms showed that the WT protein had the lowest and most stable RMSD values (2.79 ± 0.33 Å) indicating greater structural stability than either variant (Fig. 3B). R173H and, particularly, G274R exhibited increased fluctuations and overall higher RMSD values (R173H: 3.06 ± 0.37 Å; G274R: 3.12 ± 0.39 Å), suggesting that these variants destabilize the protein backbone. UDP-Gal RMSD after fitting to the protein backbone, indicated that the ligand remained more stable within the binding site in the WT system (2.35 ± 0.27 Å), whereas both variants exhibited greater fluctuations (Fig. 3C). R173H showed intermediate instability (2.87 ± 0.60 Å), while G274R displayed the highest RMSD values (3.56 ± 0.59 Å), suggesting reduced binding stability or conformational rearrangement of the ligand. Analysis of protein-ligand interactions revealed differences among WT, R173H, and G274R in complex with UDP-Gal. In the WT protein, UDP-Gal formed stable and numerous contacts, particularly with R171 and K261, which showed near-complete occupancy. The R173H variant displayed a similar interaction pattern, but with an even broader and stronger network of residues contributing to ligand binding. By contrast, the G274R mutant showed a markedly reduced interaction profile, both in the number and strength of protein-ligand contacts. In the G274R variant, only H330 reached full occupancy, with weaker but persistent interactions provided by F208, D234, and R171 (Supplemental Fig. S6A).

Overall, these MD simulation data suggest that the R173H mutation and especially the G274R mutation compromise both the structural integrity of B4GALT5 and its ability to maintain the UDP-Gal ligand within the binding site. This finding is consistent with the location of these residues in the ligand-binding pocket (Supplemental Fig. S6B) and could explain the observed functional consequences for glycosyltransferase activity.

### Functional analysis of *B4GALT5* variants

**Fig. 4:**
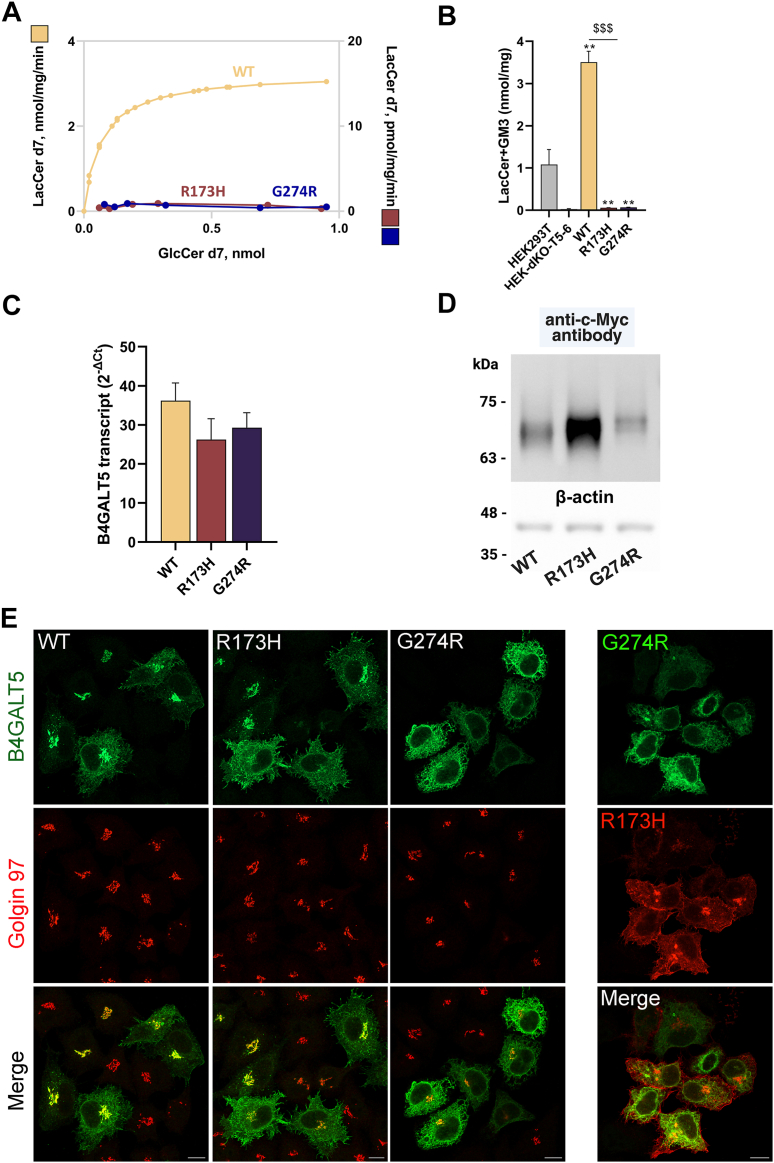
Characterization of B4GALT5 variants. A: HEK-dKO-T5-6 cells were transfected with WT B4GALT5 or patient variants (R173H and G274R) cloned in a pCDNA3 vector, and the homogenates of transfected cells were used as the enzyme source in LacCer synthase assay, as described in Figure 2. Note the two different scales used for WT and variants. The results are presented as Hanes–Woolf plots of the activity values. The reactions were obtained incubating 0.5 μg of WT homogenate protein and 60 μg of B4GALT5 variants for 30 min, which assured initial rates. B: Aliquots of the same transfected cells of A were submitted to GSL extraction and analyzed by LC-MS/MS as in Figure 2. Values are the mean ± SD for duplicate analysis performed on four independent transfections. Statistical significance refers to comparisons with HEK293T cells (∗), and to the comparison of WT ($) with both patient variants (R173H and G274R). C: RNA extracted from a third aliquot of the same transfected cells of A and B was reverse-transcribed and submitted to qPCR as in Figure 2. D: HEK-dKO-T5-6 cells were transfected with WT B4GALT5 or patient variants (R173H and G274R) cloned in a plasmid encoding a C-terminal c-Myc tag. Protein extracted from the transfected cells were separated by 10% SDS-PAGE and transferred to a nitrocellulose membrane that was blotted with anti-c-Myc antibody and visualized by chemiluminescence detection. The membrane was then stripped and blotted again with anti-β-actin antibody. Western blots performed from two independent transfections provided similar results (see Supplemental Fig. S7). E: Immunofluorescence analysis of HeLa cells transfected with WT B4GALT5 and the R173H and G274R variants tagged at the C-terminus with 3xFLAG. Cells were stained with anti-FLAG (green) and anti-golgin97 (red) antibodies. Co-expression of G274R-3xFLAG (green) and R173H-cMyc (red) is shown in the right panel. Scale bars, 10μm. In Figure 4B statistical significance was evaluated by one-way ANOVA followed by Tukey’s post hoc test.∗∗*P* < 0.01 versus HEK293T; $$$ *P* < 0.005 versus both patient’s variants (R173H, G274R).

To express WT B4GALT5 or WT B4GALT5 tagged at the C-terminus with a c-Myc sequence, B4GALT5 cDNA was placed under the control of a CMV promoter in a pcDNA3 vector. Both variants identified in the patient (c.518G>A and c.820G>A) were then introduced into both plasmids by site-directed mutagenesis, allowing expression of the corresponding R173H and G274R B4GALT5 variants in either native or C-terminal c-Myc tagged forms. LacCer synthase activity was readily detectable in homogenates obtained from HEK-dKO-T5-6 cells transfected with WT pcDNA3-B4GALT5-, using as little as 0.5 μg of total homogenate protein. By contrast, no LacCer synthase activity was detectable in homogenates from cells transfected with either pcDNA3-*B4GALT5* variant, even when up to 60 μg of total homogenate protein was used and regardless of substrate concentration (Fig. 4A). Moreover, whereas transfection with WT pcDNA3-B4GALT5 restored LacCer and GM3 levels to amounts exceeding those observed in native HEK293T cells, transfection with either pcDNA3-*B4GALT5* variant had no effect on the glycosphingolipid profile of HEK-dKO-T5-6 cells (Fig. 4B) Because mRNA levels measured in the same transfection experiments were comparable among the WT *B4GALT5* and the two variants, the variants do not appear to affect transfection efficiency, transcription, or mRNA stability (Fig. 4C). By western blotting, the apparent molecular weight of c-Myc-tagged WT B4GALT5 was similar to that of both B4GALT5 variants and corresponded to approximately 68 kDa, including the approximately 4-kDa c-Myc tag. The lower apparent molecular weight of the B4GALT5 variants observed in Figure 4D is likely a gel migration artifact because it was not reproducible in similar western blots (Supplemental Fig. S7). PNGase F treatment shifted of the B4GALT5 band to a lower molecular weight of approximately 52 kDa for both WT B4GALT5 (Supplemental Fig. S8) and the B4GALT5 variants (data not shown), suggesting that all forms are heavily N-glycosylated carrying approximately 16 kDa of N-glycans. However, protein expression levels differed between WT B4GALT5 and the variants. The band corresponding to the R173H variant was more intense than that of WT B4GALT5, whereas the G274R band was less intense (Fig. 4D). We next analyzed the intracellular distribution of the B4GALT5 variants by immunofluorescence, HeLa cells were transfected with WT B4GALT5 and the R173H and G274R variants, either individually or in combination, using constructs tagged at the C-terminus with c-Myc or 3×FLAG. WT B4GALT5 and the R173H variant localized primarily to the Golgi apparatus, with additional plasma membrane staining observed in cells expressing higher protein levels. By contrast, the G274R variant localized mainly to the endoplasmic reticulum, with only a small number of cells showing Golgi localization, and it was never detected at the plasma membrane. Co-expression of the variant proteins did not alter their subcellular localization (Fig. 4E). The plasma membrane localization of WT B4GALT5 and the R173H variant, likely resulting from saturation of Golgi retention mechanisms by overexpressed proteins, was confirmed by staining non-permeabilized HeLa cells (Supplemental Fig. S9). Furthermore, patient fibroblasts transfected with N-terminal Halo-tagged WT B4GALT5 or the R173H and G274R variants exhibited the same localization patterns as observed in HeLa cells: Golgi and plasma membrane localization for WT and R173H, and endoplasmic reticulum localization for G274R (Supplemental Fig. S10).

### Characterization of human B4GALT6

**Fig. 5:**
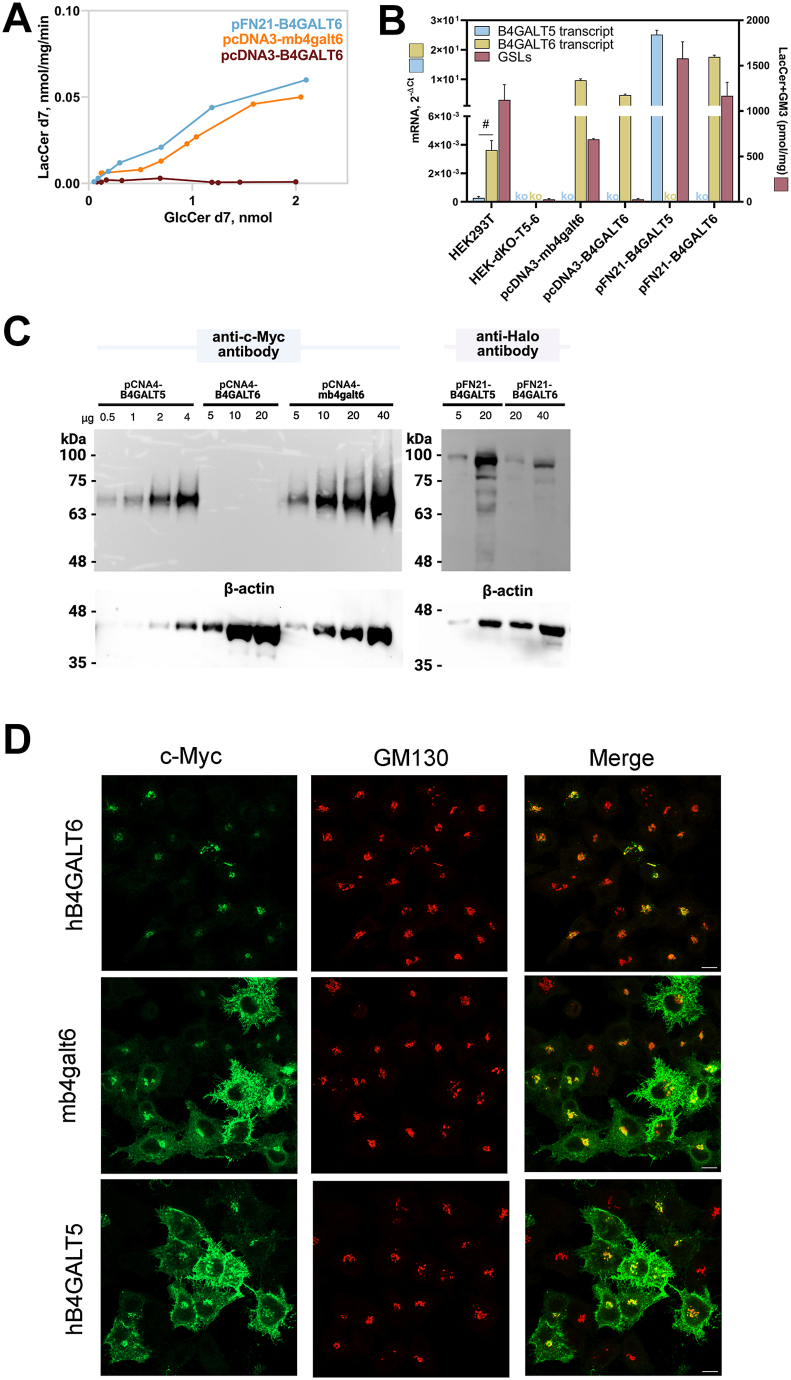
Characterization of B4GALT6. A: HEK-dKO-T5-6 were transfected with mouse or human B4GALT6 cloned in a pCDNA3 vector (pcDNA3-mb4galt6 or pcDNA3-B4GALT6, respectively), or in a pFN21 vector encoding an N-terminal Halo tag (pFN21-B4GALT6). The homogenates of transfected cells were used as the enzyme source in LacCer synthase assay, as described in Figure 2. The results are presented as Hanes–Woolf plots of the activity values. The reactions were obtained, incubating 1.0 μg of homogenate protein for 60 min, which assured initial rates. B: Aliquots of the same transfected cells as in (A), as well as non-transfected HEK-dKO-T5-6, native HEK293T cells and HEK-dKO-T5-6 transfected with pFN21-B4GALT5, were used for RNA or GSL extraction. RNA was analyzed by RT-qPCR and GSLs by LC-MS/MS as reported in Figure 2. Values labeled as ko are from transcripts in the respective KO clones, which were not suitable because the CRISPR-Cas9 procedure employed (see Materials and methods) may have removed or not the annealing sequence that the PCR primers bind to. Statistical significance (#, *P* < 0.05) refers to the comparison between B4GALT5 and B4GALT6 mRNA levels in HEK293T cells. C: HEK-dKO-T5-6 were transfected with B4GALT5 and mouse or human B4GALT6 cloned in pCNA4 vector encoding a C-terminal c-Myc tag. The same cells were also transfected with Human B4GALT5 and B4GALT6 cloned in pFN21 vector encoding an N-terminal Halo tag. Lysate prepared by RIPA were separated and visualized as in Figure 2. D: Immunofluorescence analysis of HeLa cells transfected with human B4GALT6, mouse b4galt6, and human B4GALT5 tagged at C-terminus with c-Myc and stained with anti-Myc (green) and anti-GM130 (red) antibodies. Scale bar, 10 μm. In Figure 5, paired *t* test was used to assess differences between B4GALT5 and B4GALT6 mRNA levels within the same sample. #*P* < 0.05.

Based on the results obtained from the patient’s fibroblasts and the B4GALT5 KO clone, the lack of B4GALT5 activity appears to be responsible for the significantly markedly reduced GSL levels observed in cells expressing the B4GALT5 variants compared with control cells expressing WT B4GALT5. Moreover, these effects appear to be independent of B4GALT6 transcript levels. To compare the properties of B4GALT6 and B4GALT5, we first measured LacCer synthase activity in vitro in HEK-dKO-T5-6 cells transfected with human pcDNA3-B4GALT6 or mouse pcDNA3-mb4galt6 (Fig. 5A). In contrast to the results obtained for B4GALT5, mouse b4galt6 activity did not reach saturation with increasing concentrations of the GlcCerd7 substrate, even at very high substrate concentrations. Surprisingly, no enzyme activity was detectable in HEK-dKO-T5-6 cells transfected with human B4GALT6, despite similar mRNA levels for human and mouse B4GALT6, suggesting that plasmid transfection and cDNA transcription were equally efficient for both constructs (Fig. 5B). GSL profiles were also compared between cells transfected with human pcDNA3-B4GALT6 and mouse pcDNA3-mb4galt6. These analyses showed that mouse b4galt6 was able to restore GSL synthesis to levels similar to those observed in parental HEK293T cells, although GSL synthesis remained higher in B4GALT5 transfected cells (Fig. 5B). By contrast, the activity of human B4GALT6 remained undetectable and GSLs were not restored following transfection with human pcDNA3-B4GALT6 (Fig. 5B). To investigate this discrepancy, we transfected HEK-dKO-T5-6 cells with plasmids expressing each of the three B4GALTs (human pCNA4-B4GALT5, human pCNA4-B4GALT6, and mouse pCNA4-mb4galt6) tagged at the C-terminus with c-Myc for Western blot detection. Surprisingly, human B4GALT6-myc was not detectable by Western blot. Conversely, mouse b4galt6-myc was readily detected, although at lower levels than B4GALT5-myc (Fig. 5C). These results suggested that the functional properties of human B4GALT6 could not be adequately evaluated under these experimental conditions because the protein was not detectably expressed. Transfection of pCNA4-B4GALT6 into HeLa cells yielded similar results by western blotting (data not shown). However, immunofluorescence analysis of the same cells showed that the protein was expressed and correctly localized to the Golgi apparatus, although expression levels were substantially lower than those of mouse b4galt6 and human B4GALT5 (Fig. 5D).

To address this issue, we again expressed human B4GALT6, this time using an N-terminal HaloTag for detection by western blotting (pFN21-B4GALT6). With this construct, expression of human Halo-B4GALT6 was clearly detectable by Western blot using anti-Halo antibodies (Fig. 5C). In addition, LacCer synthase activity became measurable in vitro (Fig. 5A) and transfection with pFN21-B4GALT6 restored GSL levels in HEK-dKO-T5-6 cells (Fig. 5B). The kinetics and efficiency of human Halo-B4GALT6-mediated GSL synthesis were very similar to those observed for the murine b4galt6 ortholog.

**Fig. 6:**
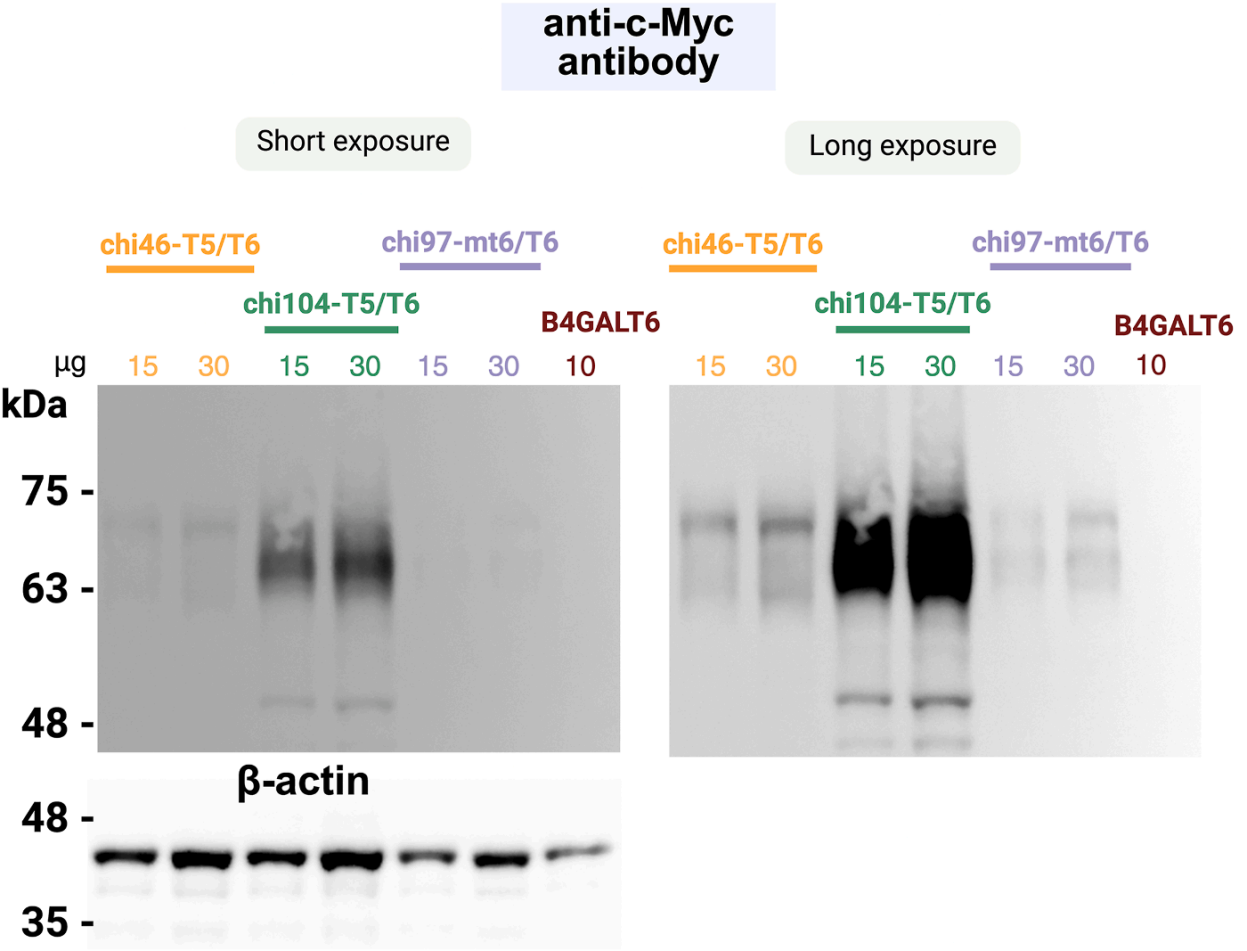
Expression of chimeric constructs of B4GALT6. Human B4GALT6 cDNA, cloned in a pCNA4 vector encoding a C-terminal c-Myc tag, was manipulated to substitute parts of the stem region with those of B4GALT5 or mouse b4galt6. Chi46-T5/T6: the first 46 amino acids of the chimera correspond to B4GALT5 protein sequence, comprising the cytosolic tail and transmembrane domain. Chi104-T5/T6: the first 101 amino acids of the chimera correspond to B4GALT5 protein sequence, comprising cytosolic tail, transmembrane domain, and stem region. Chi97-mt6/T6: the first 85 amino acids of the chimera correspond to mouse b4galt6 protein sequence, comprising cytosolic tail, transmembrane domain and stem region. For details see “Materials and Methods” and Supplemental Fig. S1. Lysate prepared from HEK-dKO-T5-6 transfected with the chimeric constructs were separated, blotted, and visualized as in Figure 3.

To further investigate why C-terminally tagged B4GALT6 was undetectable by western blotting whereas N-terminal Halo-B4GALT6 was readily detected, we repeated Western blot analyses using lysates collected at earlier time points after transfection. B4GALT5 was analyzed in parallel as a control. B4GALT5 expression was evident as early as 7 h after transfection and accumulated progressively over time. By contrast, C-terminally tagged B4GALT6 remained completely undetectable at all times examined (Supplemental Fig. S11). In our constructs, the B4GALT start codons were cloned directly into the polylinker region of pCNA4 or pcDNA3 via the *Hind*III site. We therefore generated an additional construct containing an extra 154 bp, corresponding to the entire exon 1 of B4GALT6, between the *Hind*III site and the start codon. Following transfection of this construct, B4GALT6 remained undetectable (data not shown). As a final attempt to detect C-terminally tagged B4GALT6, we generated a series of chimeric constructs. These included replacement of the sequence encoding the first 46 amino acids of B4GALT6 with the corresponding B4GALT5 sequence (chi46-T5/T6), replacement of the sequence encoding the entire stem region (104 amino acids) of B4GALT6 with that of B4GALT5 (chi104-T5/T6), and replacement of the same region with the corresponding sequence from mouse b4galt6 (chi104-mt6/T6). Surprisingly, all of these B4GALT6 chimeras were detectable, although at different levels (Fig. 6). Chi104-T5/T6 was readily detected at levels comparable to those of B4GALT5, whereas chi104-mt6/T6 appeared as two faint bands. Chi46-T5/T6 was also detected as a doublet, with bands similar to but more intense than those observed for chi104-mt6/T6. The upper band was slightly more intense than the lower band and was sensitive to PNGase F treatment (Supplemental Fig. S8), suggesting the presence of an additional glycosylation site. These results rule out the possibility of a simple technical artifact suggesting that B4GALT6 expression in transfected cells depends on the plasmid construct, not on the vector, but rather on the B4GALT6 cDNA sequence near the start codon.

## Discussion

In the present study, we found that the R173H and G274R variants in *B4GALT5* identified in a child with mild intellectual disability, microcephaly and bilateral cataract, are both functionally inactive. We also found that human B4GALT6 is less efficient as a LacCer synthase than B4GALT5. Moreover, human B4GALT6 appears to be weakly expressed despite high mRNA levels, at least in cell-based models.

Expression of WT and variant B4GALT5 constructs in HEK293T clones lacking B4GALT5, B4GALT6, or both enzymes confirmed that both B4GALT5 variants lack LacCer synthase activity following transfection, as demonstrated by a direct in vitro enzyme assay and by GSL profiling using LC-MS/MS. Whereas WT B4GALT5 showed robust activity even when minimal amounts of homogenate protein were used, no LacCer-d7 production was detectable in assays performed with either B4GALT5 variant, regardless of homogenate protein concentration or GlcCer-d7 substrate concentration. Based on GSL profiling, overexpression of WT B4GALT5 increased GSL synthesis (LacCer, GM3, and Gb3) to levels up to threefold higher than the endogenous amounts observed in parental HEK293T cells. By contrast, both B4GALT5 variants were inactive, and GSL levels remained similar to those measured in non-transfected HEK-dKO-T5-6 cells. In the same transfection experiments, mRNA levels were comparable among WT B4GALT5 and both variants. In addition, similar levels of c-Myc-tagged WT and variant B4GALT5 proteins were detected by western blotting using anti-c-Myc antibodies. Furthermore, in silico analyses predicted clear differences in conformational behavior between WT B4GALT5 and the two variants. Both R173H and G274R exhibited moderately increased RMSD values, suggesting reduced protein stability, although not to an extent that would markedly impair protein expression or prevent attainment of a stable conformation. This instability was not limited to the protein backbone. The donor substrate UDP-Gal also displayed higher and more variable RMSD values in the mutant proteins, particularly in G274R, indicating an impaired ability to stabilize the ligand within the B4GALT5 active site. Importantly, these differences were reproducible across all MD simulation replicas, supporting their biological relevance. Taken together, the in silico findings suggest that the loss of activity associated with the B4GALT5 variants results from impaired structural stability that directly affects substrate recognition and ligand binding. This interpretation is consistent with the location of the mutations within the UDP-Gal-binding pocket, where even subtle structural alterations may disrupt the precise interactions required for efficient catalysis (19).

Under flux conditions, incorporation of ^13^C_6_-galactose into GSLs was delayed and remained below control levels. Moreover, GSLs incorporated not only ^13^C_6_-galactose but also ^13^C_6_-glucose, likely because of the activity of UDP-galactose 4-epimerase, which interconverts UDP-galactose and UDP-glucose. The use of ^13^C_6_-galactose rather than ^13^C_6_-glucose remains justified because glucose contributes substantially to the synthesis of ceramide, GalNAc, and sialic acid (18). Under these experimental conditions, however, ^13^C_6_-labeled N-acetylhexosamines were incorporated only minimally into GSLs (<10% incorporation; m/z +26.104 for species containing one N-acetylhexosamine residue, such as Lc3, Lc4, or GM1a). This limited incorporation is likely due to the slower biosynthetic flux from ^13^C_6_-galactose to N-acetylhexosamine precursors, which require additional enzymatic steps and substrates, including glutamine, acetyl-CoA, and ATP. A similar limitation may affect sialic acid biosynthesis, which relies predominantly on glucose-derived rather than galactose-derived carbon sources.

Taken together, these results support the conclusion that B4GALT5 activity is lost in the patient and that GSL biosynthesis therefore depends exclusively on B4GALT6. Consistent with this interpretation, the GSL profiles detected in patient plasma and cultured primary fibroblasts were markedly altered compared with those of healthy controls. HexCer (presumably GlcCer) accumulated, whereas LacCer, GM3, and Gb3 levels were reduced by approximately 80%. Similar alterations were observed in fibroblasts and closely resembled those seen in HEK-KO-T5 cells, in which LacCer and GM3 levels were reduced by approximately 80%.

We sought to understand why B4GALT6 is unable to compensate for the loss of B4GALT5. In both HEK-KO-T5 cells and cultured primary fibroblasts, B4GALT6 transcript levels exceeded those of B4GALT5. Unfortunately, the absence of suitable anti-B4GALT6 antibodies prevented direct assessment of endogenous protein levels. Furthermore, readily detectable expression of human B4GALT6 in transfected model cells was achieved only with N-terminally tagged constructs or chimeric proteins in which the N-terminal region had been modified. Whether this finding is related to the plasmid construct, the cellular model, or previously unrecognized mechanisms regulating B4GALT6 expression in vivo remains unclear and raises a question regarding B4GALT6 expression in the context of its inability to compensate for B4GALT5 deficiency.

Our hypothesis is that when human B4GALT6 is successfully expressed, its enzymatic activity is comparable to that of the mouse ortholog but remains less efficient than that of B4GALT5, consistent with observations from knockout mouse models (4).

Because both *B4GALT5* variants expressed by the patient are inactive, the resulting reduction in overall LacCer synthase activity leads to an altered GSL profile characterized by accumulation of HexCer and an approximately 80% reduction in complex GSLs in both plasma and cultured fibroblasts. Although the human brain normally contains very high concentrations of GSLs (20), particularly gangliosides (21), it remains unclear whether a reduction of this magnitude is tolerated or pathogenic. Recent high-plex amplification stereo sequencing data available through the Human Protein Atlas indicate that B4GALT6 expression in the brain is not ubiquitous but is restricted to specific cell types, whereas B4GALT5 transcripts are detected in astrocytes, microglia, and vascular cells. If these observations are correct, some brain cell populations in the patient may lack B4GALT6 expression and therefore be completely deficient in GSL synthesis. Whether this has clinical consequences remains unknown. The patient’s clinical presentation appears relatively mild compared with that observed in ST3GAL5-CDG patients (22), who have a block in the major ganglioside biosynthetic pathway (23). Similar comparisons can be made with the heterogeneous but generally severe phenotypes reported in B4GALNT1-CDG patients (24, 25, 26) who are presumed to lack complex gangliosides (27). Early bilateral cataract provide an example of a potentially informative but nonspecific clinical feature. Cataracts are frequently associated with disorders of galactose metabolism (28, 29, 30) but are also observed in many unrelated conditions (31). Additional patients with overlapping phenotypes will therefore be required to determine whether B4GALT5 deficiency represents a novel Mendelian disorder.

In conclusion, our results suggest that patients presenting with mild cognitive and/or neurological impairment or carrying bi-allelic *B4GALT5* variants warrant further investigation of their GSL profile, which may be altered by inactive or partially inactive variants in enzymes involved in GSL biosynthesis. Glycosyltransferases are named according to the HUGO recommendations.

## Data availability

All data concerned with this study are presented within this manuscript.

## Supplemental data

This article contains supplemental data.

## Conflict of interest

The authors declare that they have no conflicts of interest with the contents of this article.
