# Supplementary material for: B4GALT5 deficiency impairs glycosphingolipid biosynthesis: a new congenital disorder of glycosylation?
Source: J Lipid Res. 2026 Jul 9;67(8):101102. doi: 10.1016/j.jlr.2026.101102 (PMC13476684; doi:10.1016/j.jlr.2026.101102)
Supplement: Supplementary file 1 [file mmc1.docx]

**Supplementary materials**

**B4GALT5 deficiency impairs glycosphingolipid biosynthesis: a new Congenital Disorder of Glycosylation?**

Linda Montavoci^1^, Michele dei Cas^1^, Anna Caretti^1^, Omar Ben Mariem^2^, Ivano Eberini^2^, Laura Giaquinto^3,4^, Alessandro De Falco^4,5,6^, Maria Antonietta de Matteis^3,4,6^, Nicola Brunetti-Pierri^4,5,6^ and Marco Trinchera^7^

^1^Department of Health Sciences, University of Milan, Milano Italy;

^2^Department of Pharmacological and Biomolecular Sciences "Rodolfo Paoletti", Università degli studi      di Milano, Milano, Italy;

^3^Department of Molecular Medicine and Medical Biotechnology, Federico II University, Napoli, Italy;

^4^Telethon Institute of Genetics and Medicine, Pozzuoli, Naples, Italy;

^5^Department of Translational Medicine, Federico II University, Napoli, Italy;

^6^Genomics and Experimental Medicine Program, Scuola Superiore Meridionale (SSM, School of Advanced Studies), Naples, Italy;

^7^DMC, University of Insubria, Varese Italy.

**Contact information for corresponding author:**

Marco Trinchera, Dipartimento di Medicina e Chirurgia, Università dell’Insubria, via JH Dunant 5, 21100 Varese, Italy, email: marco.trinchera@uninsubria.it (to whom proofs and reprints should be addressed), tel. +39 0332 397160

**Supplemental Table S1.** List of oligonucleotides used in the present study. FW, forward primer; RV, reverse primer.

| **Gene** | **Sequence** |
| --- | --- |
| **Cloning in pcDNA3** | |
| B4GALT5 FW | 5’-CGCGAAGCTTGCGATCGCCATGCGCGCCCGCCGGG-3’ |
| B4GALT5 RV | 5’-CGCGTCTAGAGTTTAAACTCAGTACTCGTTCACCTGAGCC-3’ |
| B4GALT6 FW | 5’-CGCGAAGCTTGCGATCGCCATGTCTGTGCTCAGGCGGATG-3’ |
| B4GALT6 RV | 5’-CGCGTCTAGAGTTTAAACTTAATAGTCTTCGATTGGAGCTAACTC-3’ |
| mb4galt6 FW | 5’-CGCGAAGCTTGCGATCGCCATGTCTGCGCTCAAGCGGATG-3’ |
| mb4galt6 RV | 5’-CGCGTCTAGAGTTTAAACTTAATAGTCTTCAATTGGAGCTAATTCTG-3’ |
| **Cloning in pCNA4** | |
| B4GALT5 FW | 5’-ATAGAATTCTGGCTGCAGCATGCGCGC-3’ |
| B4GALT5 RV | 5’-CGCGATATCAAGTACTCGTTCACCTGAGCC-3’ |
| B4GALT6 RV | 5’-GCGCGCGGTGACCATAGTCTTCGATTGGAGCTAACTCTG-3’ |
| mb4galt6 RV | 5’-GCGCGCGGTGACCATAGTCTTCAATTGGAGCTAATTCTGGC-3’ |
| **qPCR** | |
| B4GALT5 FW | 5’-GAGAACAATCGGTGCTCAGG-3’ |
| B4GALT5 RV | 5’-GGG CCCTTCATGGAAGGG-3’ |
| B4GALT6 FW | 5’-CCGGAAAACTTCACATACTCAC-3’ |
| B4GALT6 RV | 5’-GAACTGCCACCTTCCATCTG-3’ |
| mb4galt6 FW | 5’-GCCTTTTATGTCATCGAACAGAC-3’ |
| mb4galt6 RV | 5’-AACTCCTTATAGGGAAGAATGTAC-3’ |
| **Mutagenesis** | |
| B4GALT5 c.518G>A FW | 5’-CCCTTCCGGAACCACCACGAGCACCTC-3’ |
| B4GALT5 c.518G>A RV | 5’-GAGGTGCTCGTGGTGGTTCCGGAAGGG-3’ |
| B4GALT5 c.820G>A FW | 5’-CTTATACCGAGTTCTTTGGCAGAGTGAGTGGCTTAACAG-3’ |
| B4GALT5 c.820G>A RV | 5’-CTGTTAAGCCACTCACTCTGCCAAAGAACTCGGTATAAG-3’ |
| B4GALT6 BspEI site insertion FW | 5’-gttcaaacaacaacgtatctTccggaaaacttcacatactc-3’ |
| B4GALT6 BspEI site insertion RV | 5’-GAGTATGTGAAGTTTTCCGGAAGATACGTTGTTGTTTGAAC-3’ |
| **Chimera construction** | |
| chi46-T5/T6 FW | 5’-CGCGACGCGTTGACATTGATTATTGACTAGTT -3’ |
| chi46-T5/T6 RV | 5’-GCGCCTCGAGCTTGCATCATGAAGAGGTAGGTG-3’ |
| chi104-T5/T6 FW | 5’-CGCGACGCGTTGACATTGATTATTGACTAGTT-3’ |
| chi104-T5/T6 RV | 5’-CGCGCGTCCGGAAGAAATGTTGTAGTTTGCAGGAAG-3’ |
| chi87-mt6/T6 FW | 5’-CGCGACGCGTTGACATTGATTATTGACTAGTT-3’ |
| chi87-mt6/T6 RV | 5’-CGCGCGTCCGGAAGATACGTTGTTGTTTGAACAAG-3’ |

**Supplemental Table S2.** MRM transitions and MS/MS parameters for glycosphingolipid internal standards and endogenous species. The table reports [M+H]⁺, major product ions, declustering potential (DP), and collision energy (CE) for HexCer, LacCer, Gb3, and GM3 species with 16:0-24:0 fatty‑acyl chains. GlcCer 12:0 was used as internal standard for hexosylceramide, LacCer 12:0 for lactosylceramide and Gb3, and GM1 17:0 for gangliosides.

| **Name** | **[MH]+** | **MS/MS 1** | **MS/MS 2** | **DP (eV)** | **CE1 (eV)** |
| --- | --- | --- | --- | --- | --- |
| *IS GlcCer 12* | *644.51* | *264.26* |  | *40* | *50* |
| *IS LacCer 12* | *806.56* | *264.26* |  | *40* | *50* |
| *IS GM1 17* | *766.90 (*)* | *264.26* |  | *50* | *40* |
| HexCer 16 | 700.57 | 264.26 | 520.51 | 40 | 50 |
| HexCer 18 | 728.60 | 264.26 | 548.54 | 40 | 50 |
| HexCer 18:1 | 726.59 | 264.26 | 546.54 | 40 | 50 |
| HexCer 20 | 756.64 | 264.26 | 576.57 | 40 | 50 |
| HexCer 22 | 784.67 | 264.26 | 604.60 | 40 | 50 |
| HexCer 24 | 812.70 | 264.26 | 632.63 | 40 | 50 |
| HexCer 24:1 | 810.69 | 264.26 | 630.62 | 40 | 50 |
| LacCer 16 | 862.62 | 264.26 | 520.51 | 50 | 50 |
| LacCer 18 | 890.66 | 264.26 | 548.54 | 50 | 50 |
| LacCer 18:1 | 888.64 | 264.26 | 546.54 | 50 | 50 |
| LacCer 20 | 918.69 | 264.26 | 576.57 | 50 | 50 |
| LacCer 22 | 946.72 | 264.26 | 604.60 | 50 | 50 |
| LacCer 24 | 974.76 | 264.26 | 632.63 | 50 | 50 |
| LacCer 24:1 | 972.74 | 264.26 | 630.62 | 50 | 50 |
| GM3 16 | 1153.72 | 264.26 | 274.09 | 60 | 60 |
| GM3 18 | 1181.75 | 264.26 | 274.09 | 60 | 60 |
| GM3 18:1 | 1179.74 | 264.26 | 274.09 | 60 | 60 |
| GM3 20 | 1209.78 | 264.26 | 274.09 | 60 | 60 |
| GM3 22 | 1237.81 | 264.26 | 274.09 | 60 | 60 |
| GM3 24 | 1265.85 | 264.26 | 274.09 | 60 | 60 |
| GM3 24:1 | 1263.83 | 264.26 | 274.09 | 60 | 60 |
| Gb3 16 | 1024.68 | 264.26 | 520.51 | 60 | 60 |
| Gb3 18 | 1052.71 | 264.26 | 548.54 | 60 | 60 |
| Gb3 18:1 | 1050.69 | 264.26 | 546.54 | 60 | 60 |
| Gb3 20 | 1080.74 | 264.26 | 576.57 | 60 | 60 |
| Gb3 22 | 1108.78 | 264.26 | 604.60 | 60 | 60 |
| Gb3 24 | 1136.81 | 264.26 | 632.63 | 60 | 60 |
| Gb3 24:1 | 1134.79 | 264.26 | 630.62 | 60 | 60 |

(*) [M+2H]2+

**Supplemental Table S3**. MRM transitions for ^13^C‑galactose‑labeled glycosphingolipids. The table reports the exact mass of the protonated molecular ion [M+H]⁺ for the natural species, the expected mass shift generated by the incorporation of a ^13^C‑hexose (gal and/or glc), and the corresponding [M+H]⁺ of the labelled analogue. Transitions are shown for HexCer, LacCer, Gb3, and GM3 bearing either 16:0 or 24:0 fatty‑acyl chains; the common fragment at m/z 264.27 corresponds to the sphingoid‑base‑derived ion, whereas other fragments (e.g. for C16:0 m/z 688.58 and 856.65; C24:0 m/z 968.78 and 800.72) reflect sequential losses from the glycan moiety and thus are specific for the labelled species. For DP and CE, the values of the native lipids were used (see Supplemetal Table S2). Below is reported the schematic representation of GM3, GM1a, and LacCer structures showing the ^13^C isotopic labelling.

| **Name** | **[MH]+ unlabelled** | **Mass shift (Da)** | **[MH]+**  **labelled** | **MS/MS labelled** | | | |
| --- | --- | --- | --- | --- | --- | --- | --- |
| HexCer 16 | 700.5722 | 6.024 | 706.5962 | 264.26 | 520.50 |  |  |
| HexCer 24 | 812.6974 | 6.024 | 818.7214 | 264.26 | 632.63 |  |  |
| LacCer 16 | 862.625 | 12.048 | 874.6647 | 264.26 | 520.50 | 688.58 | 856.65 |
| LacCer 24 | 974.7502 | 12.048 | 986.7982 | 264.26 | 632.63 | 968.78 | 800.72 |
| Gb3 16 | 1024.6778 | 18.072 | 1042.73 | 264.26 | 520.50 | 688.58 | 856.65 |
| Gb3 24 | 1136.803 | 18.072 | 1154.875 | 264.26 | 632.63 | 968.78 | 800.72 |
| GM3 16 | 1153.7204 | 12.048 | 1165.7684 | 264.26 | 274.09 | 688.58 | 856.65 |
| GM3 24 | 1265.8456 | 12.048 | 1277.8936 | 264.26 | 274.09 | 968.78 | 800.72 |

**Supplemental Figure S1**

**Figure S1.** Sequences of B4GALT6 chimeras. (A) Aminoacid sequences of the stem region of B4GALT6 chimeras. Chi46T5/T6: the first 46 aminoacids are those of B4GALT5; chi104T5/T6: the first 104 aminoacids are those of B4GALT5; chi97mt6/T5: the first 97 aminoacids are those of murine b4galt6. (B) Comparison between the aminoacid sequences of B4GALT5, B4GALT6, and mouse b4galt6. Full circles denote aminoacids identical in human and mouse B4GALT6 but different in B4GALT5; asterisks denote aminoacids identical in human B4GALT5 and B4GALT6 but different in mouse bgalt6; the diamond denotes aminoacids identical in B4GALT5 and mouse b4galt6 but different in B4GALT6; the red cross denotes aminoacids different between all three B4GALTs. (C) Comparison of the corresponding DNA sequences.

**Supplemental Figure S2**

(**A**)

```
pFN21-Halo-B4GALT5
      	ATGGCAGAAATCGGTACTGGCTTTCCATTCGACCCCCATTATGTGGAAGTCCTGGGCGAGCGCATGCACTACGTC	75
GATGTTGGTCCGCGCGATGGCACCCCTGTGCTGTTCCTGCACGGTAACCCGACCTCCTCCTACGTGTGGCGCAAC
ATCATCCCGCATGTTGCACCGACCCATCGC0TGCATTGCTCCAGACCTGATCGGTATGGGCAAATCCGACAAACC
AGACCTGGGTTATTTCTTCGACGACCACGTCCGCTTCATGGATGCCTTCATCGAAGCCCTGGGTCTGGAAGAGGT	300
CGTCCTGGTCATTCACGACTGGGGCTCCGCTCTGGGTTTCCACTGGGCCAAGCGCAATCCAGAGCGCGTCAAAGG
TATTGCATTTATGGAGTTCATCCGCCCTATCCCGACCTGGGACGAAGGCCAGAATTTGCCCGCGAGACCTTCCAG
GCCTTCCGCACCACCGACGTCGGCCGCAAGCTGATCATCGATCAGAACGTTTTTATCGAGGGTACGCTGCCGATG
GGTGTCGTCCGCCCGCTGACTGAAGTCGAGATGGACCATTACCGCGAGCCGTTCCTGAATCCTGTTGACCGCGAG	600
CCACTGTGGCGCTTCCCAAACGAGCTGCCAATCGCCGGTGAGCCAGCGAACATCGTCGCGCTGGTCGAAGAATAC
ATGGACTGGCTGCACCAGTCCCCTGTCCCGAAGCTGCTGTTCTGGGGCACCCCAGGCGTTCTGATCCCACCGGCC
GAAGCCGCTCGCCTGGCCAAAAGCCTGCCTAACTGCAAGGCTGTGGACATCGGCCCGGGTCTGAATCTGCTGCAA
GAAGACAACCCGGACCTGATCGGCAGCGAGATCGCGCGCTGGCTGTCGACGCTCGAGATTTCCGGCGAGCCAACC	900

pCNA4-B4GALT5           -----------------------GAATTCTGGCTGCAGCatgcgcgcccgccgggggctg	37
pFN21-Halo-B4GALT5      ACTGAGGATCTGTACTTTCAGAGCGATAACGCGATCGCCatgcgcgcccgccgggggctg	960
pcDNA3-B4GALT5          ------------------------AAGCTTGCGATCGCCatgcgcgcccgccgggggctg	36

pCNA4-B4GALT5           ctgcggctgccgcgccgctcgctgctcgccgcgctcttcttcttttctctctcgtcctcg	97
pFN21-Halo-B4GALT5      ctgcggctgccgcgccgctcgctgctcgccgcgctcttcttcttttctctctcgtcctcg	1020
pcDNA3-B4GALT5          ctgcggctgccgcgccgctcgctgctcgccgcgctcttcttcttttctctctcgtcctcg	96

pCNA4-B4GALT5           ctgctgtacttcgtctatgtggcgcccggcatagtgaacacctacctcttcatgatgcaa	157
pFN21-Halo-B4GALT5      ctgctgtacttcgtctatgtggcgcccggcatagtgaacacctacctcttcatgatgcaa	1080
pcDNA3-B4GALT5          ctgctgtacttcgtctatgtggcgcccggcatagtgaacacctacctcttcatgatgcaa	156

pCNA4-B4GALT5           gcccaaggcattctgatccgggacaacgtgagaacaatcggtgctcaggtttatgagcag	217
pFN21-Halo-B4GALT5      gcccaaggcattctgatccgggacaacgtgagaacaatcggtgctcaggtttatgagcag	1140
pcDNA3-B4GALT5          gcccaaggcattctgatccgggacaacgtgagaacaatcggtgctcaggtttatgagcag	216

pCNA4-B4GALT5           gtgcttcggagtgcttatgccaagaggaacagcagtgtaaatgactcagattatcctctt	277
pFN21-Halo-B4GALT5      gtgcttcggagtgcttatgccaagaggaacagcagtgtaaatgactcagattatcctctt	1200
pcDNA3-B4GALT5          gtgcttcggagtgcttatgccaagaggaacagcagtgtaaatgactcagattatcctctt	276

pCNA4-B4GALT5           gacttgaaccacagtgaaaccttcctgcaaactacaacatttcttcctgaagacttcacc	337
pFN21-Halo-B4GALT5      gacttgaaccacagtgaaaccttcctgcaaactacaacatttcttcctgaagacttcacc	1260
pcDNA3-B4GALT5          gacttgaaccacagtgaaaccttcctgcaaactacaacatttcttcctgaagacttcacc	336

pCNA4-B4GALT5           tactttgcaaaccatacctgccctgaaagactcccttccatgaagggcccaatagacata	397
pFN21-Halo-B4GALT5      tactttgcaaaccatacctgccctgaaagactcccttccatgaagggcccaatagacata	1320
pcDNA3-B4GALT5          tactttgcaaaccatacctgccctgaaagactcccttccatgaagggcccaatagacata	396

pCNA4-B4GALT5           aacatgagtgaaattggaatggattacattcatgaactcttctccaaagacccaaccatc	457
pFN21-Halo-B4GALT5      aacatgagtgaaattggaatggattacattcatgaactcttctccaaagacccaaccatc	1380
pcDNA3-B4GALT5          aacatgagtgaaattggaatggattacattcatgaactcttctccaaagacccaaccatc	456

pCNA4-B4GALT5           aagctcggaggtcactggaagccttctgattgcatgcctcggtggaaggtggcgatcctt	517
pFN21-Halo-B4GALT5      aagctcggaggtcactggaagccttctgattgcatgcctcggtggaaggtggcgatcctt	1440
pcDNA3-B4GALT5          aagctcggaggtcactggaagccttctgattgcatgcctcggtggaaggtggcgatcctt	516

pCNA4-B4GALT5           atccccttccggaaccgccacgagcacctcccagtcctgttcagacacctgcttcccatg	577
pFN21-Halo-B4GALT5      atccccttccggaaccgccacgagcacctcccagtcctgttcagacacctgcttcccatg	1500
pcDNA3-B4GALT5          atccccttccggaaccgccacgagcacctcccagtcctgttcagacacctgcttcccatg	576

pCNA4-B4GALT5           ctccagcgccagcgcttgcagtttgcattttatgtggttgaacaagttggtacccaaccc	637
pFN21-Halo-B4GALT5      ctccagcgccagcgcttgcagtttgcattttatgtggttgaacaagttggtacccaaccc	1560
pcDNA3-B4GALT5          ctccagcgccagcgcttgcagtttgcattttatgtggttgaacaagttggtacccaaccc	636

pCNA4-B4GALT5           tttaatcgagccatgcttttcaacgttggctttcaagaggcaatgaaagacttggattgg	697
pFN21-Halo-B4GALT5      tttaatcgagccatgcttttcaacgttggctttcaagaggcaatgaaagacttggattgg	1620
pcDNA3-B4GALT5          tttaatcgagccatgcttttcaacgttggctttcaagaggcaatgaaagacttggattgg	696

pCNA4-B4GALT5           gactgtttgatttttcatgatgtagatcacataccggaaagtgatcgcaactattatgga	757
pFN21-Halo-B4GALT5      gactgtttgatttttcatgatgtagatcacataccggaaagtgatcgcaactattatgga	1680
pcDNA3-B4GALT5          gactgtttgatttttcatgatgtagatcacataccggaaagtgatcgcaactattatgga	756

pCNA4-B4GALT5           tgtggacagatgccgaggcattttgcaaccaaattggataagtatatgtatctgcttcct	817
pFN21-Halo-B4GALT5      tgtggacagatgccgaggcattttgcaaccaaattggataagtatatgtatctgcttcct	1740
pcDNA3-B4GALT5          tgtggacagatgccgaggcattttgcaaccaaattggataagtatatgtatctgcttcct	816

pCNA4-B4GALT5           tataccgagttctttggcggagtgagtggcttaacagtggaacaatttcggaaaatcaat	877
pFN21-Halo-B4GALT5      tataccgagttctttggcggagtgagtggcttaacagtggaacaatttcggaaaatcaat	1800
pcDNA3-B4GALT5          tataccgagttctttggcggagtgagtggcttaacagtggaacaatttcggaaaatcaat	876

pCNA4-B4GALT5           ggctttcctaatgctttctggggttggggtggagaagatgacgacctctggaacagagta	937
pFN21-Halo-B4GALT5      ggctttcctaatgctttctggggttggggtggagaagatgacgacctctggaacagagta	1860
pcDNA3-B4GALT5          ggctttcctaatgctttctggggttggggtggagaagatgacgacctctggaacagagta	936

pCNA4-B4GALT5           cagaatgcaggctattctgtgagccggccagagggtgacacaggaaagtacaagtccatt	997
pFN21-Halo-B4GALT5      cagaatgcaggctattctgtgagccggccagagggtgacacaggaaagtacaagtccatt	1920
pcDNA3-B4GALT5          cagaatgcaggctattctgtgagccggccagagggtgacacaggaaagtacaagtccatt	996

pCNA4-B4GALT5           cctcatcaccatcgaggagaagtccagtttcttggaaggtatgctctgctgaggaagtca	1057
pFN21-Halo-B4GALT5      cctcatcaccatcgaggagaagtccagtttcttggaaggtatgctctgctgaggaagtca	1980
pcDNA3-B4GALT5          cctcatcaccatcgaggagaagtccagtttcttggaaggtatgctctgctgaggaagtca	1056

pCNA4-B4GALT5           aaagaacggcaagggctggatggcctcaacaacctgaactactttgcaaacatcacatac	1117
pFN21-Halo-B4GALT5      aaagaacggcaagggctggatggcctcaacaacctgaactactttgcaaacatcacatac	2040
pcDNA3-B4GALT5          aaagaacggcaagggctggatggcctcaacaacctgaactactttgcaaacatcacatac	1116

pCNA4-B4GALT5           gacgccttgtataaaaacataactgtcaacctgacacccgagctggctcaggtgaacgag	1177
pFN21-Halo-B4GALT5      gacgccttgtataaaaacataactgtcaacctgacacccgagctggctcaggtgaacgag	2100
pcDNA3-B4GALT5          gacgccttgtataaaaacataactgtcaacctgacacccgagctggctcaggtgaacgag	1176

pCNA4-B4GALT5           tacTTGATATCCAGCACAGTGGCGGCCGCTCGAGGTCACCCATTGGAACAAAAACTCATC	1237
pFN21-Halo-B4GALT5      tactga------------------------------------------------------	2106
pcDNA3-B4GALT5          tactga------------------------------------------------------	1182

pCNA4-B4GALT5           TCAGAAGAGGATCTGAATATGCATACCGGTCATCATCACCATCACCATTGA	1288
pFN21-Halo-B4GALT5      ---------------------------------------------------	2106
pcDNA3-B4GALT5          ---------------------------------------------------	1182
```

(**B**)

```
pFN21-B4GALT6       ATGGCAGAAATCGGTACTGGCTTTCCATTCGACCCCCATTATGTGGAAGTCCTGGGCGAG	60
CGCATGCACTACGTCGATGTTGGTCCGCGCGATGGCACCCCTGTGCTGTTCCTGCACGGTAACCCGACCTCCTCCTACGT
GTGGCGCAACATCATCCCGCATGTTGCACCGACCCATCGCTGCATTGCTCCAGACCTGATCGGTATGGGCAAATCCGACA
AACCAGACCTGGGTTATTTCTTCGACGACCACGTCCGCTTCATGGATGCCTTCATCGAAGCCCTGGGTCTGGAAGAGGTC	300
GTCCTGGTCATTCACGACTGGGGCTCCGCTCTGGGTTTCCACTGGGCCAAGCGCAATCCAGAGCGCGTCAAAGGTATTGC
ATTTATGGAGTTCATCCGCCCTATCCCGACCTGGGACGAATGGCCAGAATTTGCCCGCGAGACCTTCCAGGCCTTCCGCA
CCACCGACGTCGGCCGCAAGCTGATCATCGATCAGAACGTTTTTATCGAGGGTACGCTGCCGATGGGTGTCGTCCGCCCG	540
CTGACTGAAGTCGAGATGGACCATTACCGCGAGCCGTTCCTGAATCCTGTTGACCGCGAGCCACTGTGGCGCTTCCCAAA
CGAGCTGCCAATCGCCGGTGAGCCAGCGAACATCGTCGCGCTGGTCGAAGAATACATGGACTGGCTGCACCAGTCCCCTG
TCCCGAAGCTGCTGTTCTGGGGCACCCCAGGCGTTCTGATCCCACCGGCCGAAGCCGCTCGCCTGGCCAAAAGCCTGCCT	780

pFN21-B4GALT6       AACTGCAAGGCTGTGGACATCGGCCCGGGTCTGAATCTGCTGCAAGAAGACAACCCGGAC	840
pCNA4[-154]B4GALT6 AAGCTTCTCTTTTCCGCTCGGGCCCTCTCCCCAGAGCCTCCCCGCGCGGACCCCGCTGCAGG	62

pFN21-B4GALT6       CTGATCGGCAGCGAGATCGCGCGCTGGCTGTCGACGCTCGAGATTTCCGGCGAGCCAACC	900
pCNA4[-154]B4GALT6  TCTCGGGTTCTCAGCACATTTATGGAAGTTTAGGGCCTGGACAGTGGCCCCGAGTCCGGC	122

pcDNA3B4GALT5       ------------------------AAGCTTGCGATCGCCatgcgcgcccgccgggggctg	36
Chi104-T5/T6        ------------------------AAGCTTGCGATCGCCatgcgcgcccgccgggggctg	36
Chi46-T5/T6         ------------------------AAGCTTGCGATCGCCatgcgcgcccgccgggggctg	36
pFN21-B4GALT6       ACTGAGGATCTGTACTTTCAGAGCGATAACGCGATCGCCatgtctgtgctcaggcggatg	960
pCNA4[-154]B4GALT6  CTGAGAGCGCAGCCTGGGCTGCTGGCAGGGAAGAGGAAGATGTCTGTGCTCAGGCGGATG	182
pCNA4-B4GALT6       ------------------------AAGCTTGCGATCGCCATGTCTGTGCTCAGGCGGATG	36
pcDNA3-B4GALT6      ------------------------AAGCTTGCGATCGCCatgtctgtgctcaggcggatg	36

pcDNA3B4GALT5       ctgcggctgccgcgccgctcgctgctcgccgcgctcttcttcttttctctctcgtcctcg	96
Chi104-T5/T6        ctgcggctgccgcgccgctcgctgctcgccgcgctcttcttcttttctctctcgtcctcg	96
Chi46-T5/T6         ctgcggctgccgcgccgctcgctgctcgccgcgctcttcttcttttctctctcgtcctcg	96
pFN21-B4GALT6       atgcgggtttccaatcgctctctcctcgccttcatcttcttcttctccctctcttcgtcc	1020
pCNA4[-154]B4GALT6  ATGCGGGTTTCCAATCGCTCTCTCCTCGCCTTCATCTTCTTCTTCTCCCTCTCTTCGTCC	242
pCNA4-B4GALT6       ATGCGGGTTTCCAATCGCTCTCTCCTCGCCTTCATCTTCTTCTTCTCCCTCTCTTCGTCC	96
pcDNA3-B4GALT6      atgcgggtttccaatcgctctctcctcgccttcatcttcttcttctccctctcttcgtcc	96

pcDNA3B4GALT5       ctgctgtacttcgtctatgtggcgcccggcatagtgaacacctacctcttcatgatgcaa	156
Chi104-T5/T6        ctgctgtacttcgtctatgtggcgcccggcatagtgaacacctacctcttcatgatgcaa	156
Chi46-T5/T6         ctgctgtacttcgtctatgtggcgcccggcatagtgaacacctacctcttcatgatgcaa	156
pFN21-B4GALT6       tgtctgtacttcatctatgtggccccaggcatcgccaacacatatctctttatggtacaa	1080
pCNA4[-154]B4GALT6  TGTCTGTACTTCATCTATGTGGCCCCAGGCATCGCCAACACATATCTCTTTATGGTACAA	302
pCNA4-B4GALT6       TGTCTGTACTTCATCTATGTGGCCCCAGGCATCGCCAACACATATCTCTTTATGGTACAA	156
pcDNA3-B4GALT6      tgtctgtacttcatctatgtggccccaggcatcgccaacacatatctctttatggtacaa	156

pcDNA3B4GALT5       gcccaaggcattctgatccgggacaacgtgagaacaatcggtgctcaggtttatgagcag	216
Chi104-T5/T6        gcccaaggcattctgatccgggacaacgtgagaacaatcggtgctcaggtttatgagcag	216
Chi46-T5/T6         gctcgaggtataatgttgagagaaaatgtgaaaacaataggtcatatgatcaggctgtac	216
pFN21-B4GALT6       gctcgaggtataatgttgagagaaaatgtgaaaacaataggtcatatgatcaggctgtac	1140
pCNA4[-154]B4GALT6  GCTCGAGGTATAATGTTGAGAGAAAATGTGAAAACAATAGGTCATATGATCAGGCTGTAC	362
pCNA4-B4GALT6       GCTCGAGGTATAATGTTGAGAGAAAATGTGAAAACAATAGGTCATATGATCAGGCTGTAC	216
pcDNA3-B4GALT6      gctcgaggtataatgttgagagaaaatgtgaaaacaataggtcatatgatcaggctgtac	216

pcDNA3B4GALT5       gtgcttcggagtgcttatgccaagaggaacagcagtgtaaatgactcagattatcctctt	276
Chi104-T5/T6        gtgcttcggagtgcttatgccaagaggaacagcagtgtaaatgactcagattatcctctt	276
Chi46-T5/T6         a------------------caaataaaaacagtacgctcaacggtacagattatcccgaa	258
pFN21-B4GALT6       a------------------caaataaaaacagtacgctcaacggtacagattatcccgaa	1182
pCNA4[-154]B4GALT6  A------------------CAAATAAAAACAGTACGCTCAACGGTACAGATTATCCCGAA	404
pCNA4-B4GALT6       A------------------CAAATAAAAACAGTACGCTCAACGGTACAGATTATCCCGAA	258
pcDNA3-B4GALT6      a------------------caaataaaaacagtacgctcaacggtacagattatcccgaa	258

pcDNA3B4GALT5       gacttgaaccacagtgaaaccttcctgcaaactacaacatttctTcctgaagacttcacc	336
Chi104-T5/T6        gacttgaaccacagtgaaaccttcctgcaaactacaacatttctTccggaaaacttcACA	336
Chi46-T5/T6         ggcaataattcaagtgattatcttgttcaaacaacaacgtatctcccggaaaacttcaca	318
pFN21-B4GALT6       ggcaataattcaagtgattatcttgttcaaacaacaacgtatctcccggaaaacttcaca	1242
pCNA4[-154]B4GALT6  GGCAATAATTCAAGTGATTATCTTGTTCAAACAACAACGTATCTCCCGGAAAACTTCACA	464
pCNA4-B4GALT6       GGCAATAATTCAAGTGATTATCTTGTTCAAACAACAACGTATCTCCCGGAAAACTTCACA	318
pcDNA3-B4GALT6      ggcaataattcaagtgattatcttgttcaaacaacaacgtatctcccggaaaacttcaca	318

All B4GALT6         TACTCACCATACCTCCCCTGTCCAGAAAAGCTGCCTTATATGCGAGGATTCCTCAATGTC
Constructs          AATGTAAGCGAAGTCAGTTTTGATGAAATTCATCAACTTTTCTCCAAGGATTTAGATATT
                    GAGCCAGGGGGTCATTGGAGGCCAAAAGACTGTAAACCCAGATGGAAGGTGGCAGTTCTC
                    ATTCCTTTCCGTAATCGCCATGAACATCTTCCAATTTTTTTCTTACATCTGATTCCAATG
                    CTCCAGAAGCAGCGGCTGGAATTTGCGTTTTATGTCATTGAACAGACTGGCACACAACCT
                    TTTAACCGTGCGATGCTTTTCAATGTGGGCTTCAAAGAGGCCATGAAAGACAGTGTCTGG
                    GACTGTGTAATCTTCCACGATGTGGATCATCTACCTGAAAATGACCGGAACTATTACGGA
                    TGTGGAGAAATGCCACGTCATTTTGCTGCAAAGCTGGATAAATACATGTATATTCTTCCA
                    TATAAAGAATTTTTTGGTGGTGTAAGTGGGCTGACAGTGGAACAATTTAGAAAGATCAAT
                    GGTTTTCCTAATGCCTTCTGGGGATGGGGAGGAGAAGATGATGACCTTTGGAACAGAGTT
                    CACTATGCTGGATATAATGTAACCAGACCAGAGGGAGACTTAGGAAAATACAAGTCAATT
                    AAGGAGCGTCAGTACATCGATGGACTGAACAATTTAATATATAGGCCAAAAATACTGGTT
     GATAGGTTGTATACAAACATATCTGTAAACCTCATGCCAGAGTTAGCTCCAATCGAAGAC

Chi104-T5/T6        TATGGTCACCCATTCGAACAAAAACTCATCTCAGAAGAGGATCTGAATATGCATACCGGT	1245
Chi46-T5/T6         TATGGTCACCCATTCGAACAAAAACTCATCTCAGAAGAGGATCTGAATATGCATACCGGT	1227
pFN21-B4GALT6       tattaa------------------------------------------------------	2088
pCNA4[-154]B4GALT6  TATGGTCACCCATTCGAACAAAAACTCATCTCAGAAGAGGATCTGAATATGCATACCGGT	1364
pCNA4-B4GALT6       TATGGTCACCCATTCGAACAAAAACTCATCTCAGAAGAGGATCTGAATATGCATACCGGT	1227
pcDNA3-B4GALT6      tattaa------------------------------------------------------	1173

Chi104-T5/T6        CATCATCACCATCACCATTGA	1259
Chi46-T5/T6         CATCATCACCATCACCATTGA	1239
pFN21-B4GALT6       ---------------------	2088
pCNA4[-154]B4GALT6  CATCATCACCATCACCATTGA	1385
pCNA4-B4GALT6       CATCATCACCATCACCATTGA	1239
```

(**C**)

```
Chi-97mT6/T6      AAGCTTGCGATCGCCATGTCTGCGCTCAAGCGGATGATGCGGGTCTCCAATCGCTCTCTG	60
pCNA4-mb4galt6    AAGCTTGCGATCGCCATGTCTGCGCTCAAGCGGATGATGCGGGTCTCCAATCGCTCTCTG	60
pcDNA3-mb4galt6   AAGCTTGCGATCGCCatgtctgcgctcaagcggatgatgcgggtctccaatcgctctctg	60

Chi-97mT6/T6      ATTGCCTTCATCTTCTTCTTCTCCCTCTCCACGTCCTGTCTCTACTTCATCTATGTGGCT	120
PCNA4-mb4galt6    ATTGCCTTCATCTTCTTCTTCTCCCTCTCCACGTCCTGTCTCTACTTCATCTATGTGGCT	120
pcDNA3-mb4galt6    attgccttcatcttcttcttctccctctccacgtcctgtctctacttcatctatgtggct	120

Chi-97mT6/T6      CCAGGCATCGCCAACACGTACCTCTTTATGGTACAAGCTAGAGGTATCATGCTGAGAGAG	180
PCNA4-mb4galt6    CCAGGCATCGCCAACACGTACCTCTTTATGGTACAAGCTAGAGGTATCATGCTGAGAGAG	180
pcDNA3-mb4galt6  ccaggcatcgccaacacgtacctctttatggtacaagctagaggtatcatgctgagagag	180

Chi-97mT6/T6      AATGTGAAAACAATTGGACACATGATCAGGCTGTATACCAATAAAAACACTACGCTCAAC	240
PCNA4-mb4galt6    AATGTGAAAACAATTGGACACATGATCAGGCTGTATACCAATAAAAACACTACGCTCAAC	240
pcDNA3-mb4galt6   aatgtgaaaacaattggacacatgatcaggctgtataccaataaaaacactacgctcaac	240

Chi-97mT6/T6      GGAACAGATTATCCTGAAGGCAATAATACAAGTGACTATCTTGTTCAAACAACAACGTAT	300
PCNA4-mb4galt6    GGAACAGATTATCCTGAAGGCAATAATACAAGTGACTATCTTGTTCAAACAACAACGTAT	300
pcDNA3-mb4galt6   ggaacagattatcctgaaggcaataatacaagtgactatcttgttcaaacaacaacgtat	300

Chi-97mT6/T6      CTTCCGGAAAACTTCACATACTCACCATACCTCCCCTGTCCAGAAAAGCTGCCTTATATG	360
PCNA4-mb4galt6    CTTCCGCAAAACTTCACGTATTTGCCACACCTCCCCTGTCCAGAGAAGCTGCCTTACATG	360
pcDNA3-mb4galt6   cttccgcaaaacttcacgtatttgccacacctcccctgtccagagaagctgccttacatg	360

Chi-97mT6/T6      CGAGGATTCCTCAATGTCAATGTAAGCGAAGTCAGTTTTGATGAAATTCATCAACTTTTC	420
PCNA4-mb4galt6    CGGGGATTCCTCAGTGTCAATGTGAGTGAGATCAGTTTTGATGAAGTCCATCAGCTCTTC	420
pcDNA3-mb4galt6   cggggattcctcagtgtcaatgtgagtgagatcagttttgatgaagtccatcagctcttc	420

Chi-97mT6/T6      TCCAAGGATTTAGATATTGAGCCAGGGGGTCATTGGAGGCCAAAAGACTGTAAACCCAGA	480
PCNA4-mb4galt6    TCCAAGGACTCAGAGATTGGGCCAGGAGGCCACTGGAGGCCCAAAGACTGCAAGCCCAGA	480
pcDNA3-mb4galt6   tccaaggactcagagattgggccaggaggccactggaggcccaaagactgcaagcccaga	480

Chi-97mT6/T6      TGGAAGGTGGCAGTTCTCATTCCTTTCCGTAATCGCCATGAACATCTTCCAATTTTTTTC	540
PCNA4-mb4galt6    TGGAAGGTGGCAGTTCTCATCCCCTTCCGTAACCGCCATGAACATCTTCCGATTTTTTTC	540
pcDNA3-mb4galt6   tggaaggtggcagttctcatccccttccgtaaccgccatgaacatcttccgatttttttc	540

Chi-97mT6/T6      TTACATCTGATTCCAATGCTCCAGAAGCAGCGGCTGGAATTTGCGTTTTATGTCATTGAA	600
PCNA4-mb4galt6    CTGCACCTGATTCCGATGCTCCAGAAACAGCGGCTGGAATTTGCCTTTTATGTCATCGAA	600
pcDNA3-mb4galt6   ctgcacctgattccgatgctccagaaacagcggctggaatttgccttttatgtcatcgaa	600

Chi-97mT6/T6      CAGACTGGCACACAACCTTTTAACCGTGCGATGCTTTTCAATGTGGGCTTCAAAGAGGCC	660
PCNA4-mb4galt6    CAGACCGGCACACAACCATTCAATCGTGCGATGCTCTTTAATGTGGGTTTCAAAGAGGCC	660
pcDNA3-mb4galt6   cagaccggcacacaaccattcaatcgtgcgatgctctttaatgtgggtttcaaagaggcc	660

Chi-97mT6/T6      ATGAAAGACAGTGTCTGGGACTGTGTAATCTTCCACGATGTGGATCATCTACCTGAAAAT	720
PCNA4-mb4galt6    ATGAAAGACAGAGCCTGGGACTGTGTGATCTTCCATGATGTGGATCATCTGCCTGAAAAT	720
pcDNA3-mb4galt6   atgaaagacagagcctgggactgtgtgatcttccatgatgtggatcatctgcctgaaaat	720

Chi-97mT6/T6      GACCGGAACTATTACGGATGTGGAGAAATGCCACGTCATTTTGCTGCAAAGCTGGATAAA	780
PCNA4-mb4galt6    GATCGGAACTACTACGGATGTGGAGAGATGCCGCGGCACTTTGCAGCGAAGCTGGACAAG	780
pcDNA3-mb4galt6   gatcggaactactacggatgtggagagatgccgcggcactttgcagcgaagctggacaag	780

Chi-97mT6/T6      TACATGTATATTCTTCCATATAAAGAATTTTTTGGTGGTGTAAGTGGGCTGACAGTGGAA	840
PCNA4-mb4galt6    TACATGTACATTCTTCCCTATAAGGAGTTTTTTGGTGGGGTGAGTGGCCTGACGGTGGAG	840
pcDNA3-mb4galt6   tacatgtacattcttccctataaggagttttttggtggggtgagtggcctgacggtggag	840

Chi-97mT6/T6      CAATTTAGAAAGATCAATGGTTTTCCTAATGCCTTCTGGGGATGGGGAGGAGAAGATGAT	900
PCNA4-mb4galt6    CAATTCAGGAAGATCAACGGCTTTCCCAATGCCTTCTGGGGATGGGGAGGCGAGGACGAT	900
pcDNA3-mb4galt6   caattcaggaagatcaacggctttcccaatgccttctggggatggggaggcgaggacgat	900

Chi-97mT6/T6      GACCTTTGGAACAGAGTTCACTATGCTGGATATAATGTAACCAGACCAGAGGGAGACTTA	960
PCNA4-mb4galt6    GACCTTTGGAACAGAGTTCACTACGCCGGCTACAATGTAACCCGGCCAGAAGGGGACTTG	960
pcDNA3-mb4galt6  gacctttggaacagagttcactacgccggctacaatgtaacccggccagaaggggacttg	960

Chi-97mT6/T6      GGAAAATACAAGTCAATTCCTCATCACCATAGAGGTGAAGTCCAGTTTTTAGGACGGTAT	1020
PCNA4-mb4galt6    GGAAAATACATATCTATTCCTCATCACCATCGAGGGGAGGTACAGTTCCTAGGACGGTAT	1020
pcDNA3-mb4galt6  ggaaaatacatatctattcctcatcaccatcgaggggaggtacagttcctaggacggtat	1020

Chi-97mT6/T6      AAATTACTAAGGTATTCCAAGGAGCGTCAGTACATCGATGGACTGAACAATTTAATATAT	1080
PCNA4-mb4galt6    AAATTACTTCGGTATTCCAAGGAGCGGCAGTACATCGATGGACTGAACAATTTATTATAC	1080
pcDNA3-mb4galt6   aaattacttcggtattccaaggagcggcagtacatcgatggactgaacaatttattatac	1080

Chi-97mT6/T6      AGGCCAAAAATACTGGTTGATAGGTTGTATACAAACATATCTGTAAACCTCATGCCAGAG	1140
PCNA4-mb4galt6    ACACCCAAAATACTGGTTGATAGGCTGTATACAAATATATCTGTAAACCTCATGCCAGAA	1140
pcDNA3-mb4galt6   acacccaaaatactggttgataggctgtatacaaatatatctgtaaacctcatgccagaa	1140

Chi-97mT6/T6      TTAGCTCCAATCGAAGACTATGGTCACCCATTCGAACAAAAACTCATCTCAGAAGAGGAT	1200
PCNA4-mb4galt6    TTAGCTCCAATTGAAGACTATGGTCACCCATTGGAACAAAAACTCATCTCAGAAGAGGAT	1200
pcDNA3-mb4galt6   ttagctccaattgaagactattaa------------------------------------	1164

Chi-97mT6/T6      CTGAATATGCATACCGGTCATCATCACCATCACCATTGA	1239
PCNA4-mb4galt6    CTGAATATGCATACCGGTCATCATCACCATCACCATTGA	1244
pcDNA3-mb4galt6  ---------------------------------------	1164
```

**Figure S2**. Alignment of cDNA sequences used in this study. Restriction sites for cloning are highlighted in green (HindIII), pink (BstEII), or are underlined (SfgI). The c-Myc sequence is highlighted in yellow. (A) Alignment of human B4GALT5 cDNA sequence cloned in three vectors. (B) Alignment of human B4GALT6 cDNA sequence cloned in three vectors or inserted in two B4GALT5/B4GALT6 chimeras. The B4GALT5 cDNA sequence present in the chimeras is highlighted in grey. At codon 96, coding lysine, the C>T change forming a BspeI restriction site (TCCGGA) is highlighted in turquoise. (C) Alignment of mouse b4galt6 cDNA sequence cloned in two vectors or inserted in a mouse/human chimera. The mouse cDNA sequence present in the chimera is highlighted in grey.

**Supplemental Figure S3.**

**Supplemental Figure S3.** B4GALT5/6 mRNA in fibroblasts. B4GALT5 and B4GALT6 mRNA levels of dermal fibroblasts from six healthy individuals and from the patient are shown. Statistical significance (#) refers to the comparison between mRNA of B4GALT5 and B4GALT6 within each sample. Paired t- test was used to assess the statistical difference. # p<0.05, ## p<0.01, ### p<0.005.

**Supplemental Figure S4**

**Figure S4.** Sphingolipid neo-synthesis in fibroblasts. **(A)** Representative thin-layer chromatography (TLC) of [^3^H]-sphingosine-labeled sphingolipids after a 2-hour pulse in control and patient fibroblasts. **(B)** Quantification of radiolabeled sphingolipid species in Control (blue) and Patient (orange) fibroblasts following 1-hour and 2-hour [^3^H]-sphingosine pulses.

**Supplemental Figure S5.**

**Figure S5.** Effect of CRISPR/Cas9-mediated homology-directed repair introducing a red fluorescent protein/puromycin cassette. Total RNA was extracted from HEK293T cells and from the obtained KO clones, reverse transcribed, and submitted to PCR with the primer pairs used for cloning the full coding sequence of B3GALT5 or B4GALT6. The same reaction conditions were used but repeating PCR cycles 38 folds (see supplemental table S1 and “Material and methods”). Five μL of each amplification were spotted on an 1% agarose gel run for 1h at 90 Volts in TBE buffer. HEK-dKO-T5-6 clones 1 and 2 were obtained from HEK-KO-T5 clone 1 upon Cre plasmid transfection followed by isolation of clones returned negative under fluorescence microscopy and sensitive to puromycin, which were then submitted to B4GALT6 CRISPR/Cas9-HDR genome editing.

**Supplemental Figure S6.**

**Figure S6.**UDP-Gal binding.  **(A)** For each protein, the occupancy of interactions with UDP-Gal across all replicas was calculated as the percentage of frames in which the interaction occurs relative to the total number of frames. **(B)** A focus on the binding site is represented. WT residues G274 (pink) and R173 (light blue) are highlighted in the left panel, while the corresponding mutant residues R274 (pink) and H173 (light blue) are shown in the right panel. UDP is represented as sticks and the manganese ion as a sphere. Both structures are displayed in the same orientation.

**Supplemental Figure S7**

**Figure S7**. Characterization of B4GALT5 variants. HEK-dKO-T5-6 cells (left panel) and HeLa cells (right panel) were transfected with WT B4GALT5 or patient variants (R173H and G274R) cloned in a plasmid encoding a C-terminal c-Myc tag. Protein extracted from the transfected cells were separated by 10% SDS-PAGE and transferred to a nitrocellulose membrane that was blotted with anti-c-Myc antibody and visualized by chemiluminescence detection.

**Supplemental Figure S8**

**Figure S8.** Expression of chimeric constructs of B4GALT6. Chimeric constructs of B4GALT6 were prepared, transfected into HEK-dKO-T5-T6, and homogenate protein separated, blotted, and visualized as in Fig.6 of the main text. Some samples were deglycosylated by PNGase F treatment. The indicated plasmids were all cloned in a pCNA4 vector encoding a C-terminal c-Myc tag.

**Supplemental Figure S9.**

**Figure S9.** Plasma membrane staining of HeLa cells expressing recombinant B4GALT5. Non-permeabilized HeLa cells transfected with WT B4GALT5 or the R173H and G274R variants tagged at the C-terminus with c-Myc, and stained with anti-Myc antibody (green). Scale bars, 10μm.

**Supplemental Figure S10.**

**Figure S10**. Expression of Halo-tagged B4GALT5 in fibroblasts. Patient fibroblasts transfected with WT B4GALT5 or the R173H and G274R variants tagged at the N-terminus with a Halo tag. Cells were incubated with the fluorescent TMR ligand (red) for 15 minutes at 37˚C prior to fixation, and then stained with anti-Golgin97 (green) and anti-VAPA (grey) antibodies. Co-localization of B4GALT5 (red) and Golgi (green) is shown in the right panel. Scale bars, 10μm.

**Supplemental Figure S11.**

**Figure S11.** Time course of B4GALT5 and B4GALT6 expression. HEK-dKO-T5-6 cells were transfected with pCNA4-B4GALT5 or pCNA4-B4GALT6, two plasmids encoding a C-terminal c-Myc tag. At different times upon transfection, cells were detached, protein extracted by RIPA buffer, and 20 μg separated by 10% SDS-PAGE. The gel was transferred to a nitrocellulose membrane that was blotted with anti-c-Myc antibody and visualized by chemiluminescence detection. The membrane was then stripped and blotted again with anti-β-actin antibody.
